# Comprehensive Multiomics Analysis Identified IQGAP3 as a Potential Prognostic Marker in Pan-Cancer

**DOI:** 10.1155/2022/4822964

**Published:** 2022-09-16

**Authors:** Guoqing Wang, Xiao Zhou, Yuanyuan Li, Min Zhao, Yiming Zou, Qicheng Lu, Yugang Wu

**Affiliations:** ^1^Department of Gastrointestinal Surgery, The Third Affiliated Hospital of Soochow University, Changzhou First People's Hospital, Soochow University, Changzhou, China; ^2^Department of Gastrointestinal Surgery, Changzhou Maternal and Child Health Care Hospital, Changzhou Medical Center, Nanjing Medical University, China; ^3^Department of Urinary Surgery, The Third Affiliated Hospital of Soochow University, Changzhou First People's Hospital, Soochow University, Changzhou, China

## Abstract

**Background:**

IQGAP3 has important function in cancer progression and has become a potential therapeutic target as a transmembrane protein. But its role in tumor immunity and pan-cancer was not systematically investigated. This study evaluated the potential role of IQGAP3 and clinical significance in pan-cancer through combined multiomics analysis.

**Methods:**

From Genotype Tissue Expression (GTEx) and The Cancer Genome Atlas (TCGA) databases, transcriptomic datasets were first obtained, and from Gene Expression Omnibus (GEO), expression profiling microarray data were acquired and integrated to systematically assess the expression differences and prognostic relevance of IQGAP3 in pancreatic cancer. Immunohistochemical data were obtained from Human Protein Atlas (HPA) to assess IQGAP3 protein expression differences, and exome data from TCGA were used to analyze IQGAP3 expression in relation to tumor mutational burden (TMB), microsatellite instability (MSI), and mutation. Additionally, we also analyzed the relationship between IQGAP3 expression and immune checkpoints, mismatch repair (MMR), and IQGAP3 relationship with methylation and copy number variation based on expression profiles.

**Results:**

Microsatellite instability (MSI), immune checkpoints, mismatch repair (MMR), and tumor mutational burden (TMB) all closely interacted with IQGAP3 mRNA. In addition, detailed relationships between the immune microenvironment and IQGAP3 mRNA as well as immune cell CD4+ Th2 and myeloid-derived suppressor cells (MDSCs) were determined. Mechanistically, IQGAP3 was involved in cytoskeleton formation, T cell receptor signaling pathways, DNA damage, cell cycle, P53 pathway, Fc gamma R-mediated phagocytosis, and apoptosis.

**Conclusion:**

IQGAP3 could serve as an effective prognostic biomarker for pan-cancer immune-related therapy.

## 1. Introduction

The incidence of malignant neoplasms has increased at an alarming rate in the last decades, which can be attributed to the increase in life expectancy, changes in lifestyle habits, and the interaction between genetic factors and external factors (physical, chemical, and biological carcinogens) [1]. Pan-cancer analysis has been widely used in cancer research to reveal the common features, heterogeneity, emerging themes, and breadth of analysis of various human malignancies [2]. Pan-cancer analysis is the analysis of molecular abnormalities in various types of cancer that identifies any common features and heterogeneity in important biological processes that are in a state of dysregulation due to different cancer cell lineages. Pan-cancer analysis projects, such as the Cancer Cell Lineage Encyclopedia (CCLE) and The Cancer Genome Atlas (TCGA), are created based on the evaluation of different human cancer cell lines and tissues at the epigenomic, genomic, proteomic, and transcriptomic levels [3–5].

The GTPase isoleucine-glutamine sequence activator protein (IQGAP) is an evolutionarily conserved protein family that includes three members, IQGAP1, IQGAP2, and IQGAP3. Its main components include calmodulin homology domain (CHD), polyproline binding domain (WW), calmodulin binding protein (IQ), and GTPase activator protein-related domain (RGD). They are implicated in regulating different cellular processes, for example, intracellular signal transduction [6], cell proliferation [7, 8], cell migration [9], and cell division [10].

IQGAP3 is the newest member of this family and is located at 1q21.3, a region with a high incidence of cancer spread [10]. IQGAP3 has been shown to be overexpressed in liver cancer [11], colorectal cancer [12], and breast cancer [13]. IQGAP3 is involved in various tumor pathways, including MAPK signaling pathway [14], Ras signaling pathway [14], and TGF-*β*/Smad signaling pathway [15]. However, the potential role of IQGAP3 in various tumor types has not been fully elucidated. Therefore, a systematic analysis of IQGAP3 in pan-cancer was conducted based on multiomics data; here, IQGAP3 was determined as an effective prognostic molecular immune biomarker. In addition, the role of IQGAP3 in immunotherapy and targeted therapy may shed light on future tumor therapy.

## 2. Materials and Methods

### 2.1. Data Acquisition

We collected IQGAP3 data on various cancer samples from TCGA (http://cancergenome.nih.gov) database via the UCSC Xena platform (http://xena.ucsc.EDU) [16]. It mainly includes related clinical data, somatic mutation, and RNA sequencing of 33 cancers. Clinical data included disease-specific survival (DSS), progression-free (PFI), and overall survival (OS), disease-free (DFI) data for 33 cancer patients. Supplementary Table 1 shows the detailed abbreviations for the 33 cancer types. From GTEx (https://commonfund.nih.gov/gtex), we downloaded gene expression data of 31 different tissues. We manually retrieved m6A-related literature from PUBMED (https://pubmed.ncbi.nlm.nih.gov/) and collected m6A regulators from past literature.

### 2.2. IQGAP3 mRNA Expression in Pan-Cancer

To explore the expression levels of IQGAP3 in normal tissues and various cancer tissues, we used data from TCGA and GTEx. TIMER.2 (timer2.0; http://timer.Cistrome.org/) [17], a resource platform, allows the exploration of TCGA-based cancer-related analyses, including coexpression analysis, gene differential expression analysis, and tumor immune correlation analysis. With “gene_de” module in Tumor Immune Estimation Resource 2.0 (TIMER2.0), the differential expressions of IQGAP3 mRNA in TCGA tumors were analyzed. For statistical significance, the Wilcoxon test was used. TCGA database was not statistically convincing due to the lack of normal samples in some tumors. Therefore, normal samples from TCGA and GTEx databases were integrated to match tumor samples from TCGA database, and to respond more convincingly, we further conducted log2(x + 0.001) transformation on each expression value. We calculated differences in IQGAP3 expression between tumorous and healthy samples in each tumor using the R software (version 3.6.3) and analyzed the significance of differences using unpaired Wilcoxon rank sum and signed rank tests. These analytics were implemented via SangerBox. Statistical significance was considered if *P* < 0.05.

### 2.3. Expression of IQGAP3 at the Protein Level and Its Localization in Subcellular

The Human Protein Atlas (HPA) portal (http://www.proteinatlas.org) [18] offers information on the cellular and tissue distribution of 26,000 human proteins. Here in the database, using highly specific antibodies and using immunoassay techniques (immunohistochemistry, immunofluorescence, and immunoblotting), researchers examined each protein in detail in 48 human normal tissues and 64 cell lines [19]. With HPA, we explored the subcellular localization of IQGAP3, and, according to the tissue and pathology section, we explored the expression of IQGAP3 in protein and compared it with IQGAP3 mRNA expression.

### 2.4. Association between IQGAP3 Expression with Prognosis and Tumor Stage

To understand the association of tumor prognosis with IQGAP3, we analyzed the relationship of OS, DFI, PFI, and DSS with IQGAP3 expression in each tumor by the Kaplan-Meier survival analysis in the “survival” R package. In addition, we created forest maps using the Cox regression analysis in “survminer” and “forestplot” R packages. *P* < 0.05 was statistically significant.

We further validated the relationship between IQGAP3 and patient outcomes using the PrognoScan (http://www.prognoscan.org) [20] database. The source of data for the PrognoScan database is different from the aforementioned databases. Its data sources are mainly Gene Expression Omnibus (GEO), ArrayExpress, and individual laboratory websites. In addition, we used R packages “limma” to analyze IQGAP3 mRNA expression difference in TNM stage of various tumors. The R software ggpubr package was used to perform statistical analyses, and *P* < 0.05 was considered significant.

### 2.5. Mutation Profiles of IQGAP3 in Different Tumor Tissues

We investigated IQGAP3 mutations in the pan-cancer group through Cancer Genomics cBioPortal (http://www.cbioportal.org) [21], an open-access cancer genome database. In this study, the maftools package was used to observe genomic changes in IQGAP3 in 32 TCGA cancer types. At the same time, using a summary table of cancer types, the frequency of change for each cancer type was plotted. Furthermore, the positions of specific mutations in protein domains and IQGAP3 were explained based on mutation signatures.

### 2.6. IQGAP3 CNV Profiles in Pan-Cancer

Genomic Cancer Analysis (GSCA) (http://bioinfo.life.hust.edu.cn/web/gsca/) [22] integrates multiple TCGA omics data and RNAactDrug database. GSCA supports analyses such as methylation, pathway activity, copy number variation (CNV), drug sensitivity, and immune penetration. According to the CNV model of GSCA, heterozygous/homozygous and amplification/deletion of pan-cancer IQGAP3 gene and the correlation between IQGAP3 gene expression and CNV Spearman's difference in IQGAP3 gene and wild-type survival difference were analyzed.

### 2.7. Methylation Profile of IQGAP3 in Pan-Cancer

We assessed differential methylation of IQGAP3 between normal and tumorous samples. Spearman's correlation between methylation and IQGAP3 mRNA expression through the GSCA database and survival comparison (OS and DSS) between IQGAP3 hypermethylation with hypomethylation in different cancer types were assessed. By using the cor.test function, the coexpression patterns of IQGAP3 expression and M6A-related genes were analyzed, and Spearman's correlation analysis was performed.

### 2.8. IQAGP3 Expression and Immunotherapy, Immune Checkpoints and Tumor Microenvironment

Based on the data in TCGA database, coexpression analysis of IQGAP3 mRNA and genes encoding MHC, mismatch repair genes (MMR) genes, immune activation, chemokine receptor protein-related genes, and chemokines were explored and plotted heatmap. The “limma” package was used for the coexpression analysis using human-related tests, and the “reshape2” and “RColorBrewer” packages were used for visualization.

Microsatellite instability (MSI) status and tumor mutational burden (TMB) are biomarkers for evaluating immunotherapy and selecting high-quality immunotherapy groups [23]. We analyzed the relationship between TMB, IQGAP3 mRNA expression, and MSI by Spearman's correlation and visualized these results using the “fmsb” package based on TCGA somatic mutation data. Furthermore, to explore the correlation of IQGAP3 mRNA with the immune scores, matrix scores and immune microenvironment were calculated using the “estimate” and “limma” packages. For correlation coefficient calculations, Spearman's test was used.

### 2.9. IQGAP3 mRNA Expression Level and Immune Cell Infiltration

To further explore the relationship between immune infiltration and IQGAP3 in the tumor microenvironment. Based on TCGA data, to investigate the potential relationship between different levels of immune cell infiltration and IQGAP3 gene expression in different tumor types, we utilized XCELL algorithms, the TMER2 database, and TIDE.

### 2.10. Enrichment Analysis

We screened 49 experimentally validated IQGAP3-binding proteins based on the String ((https://string-db.org/) [24] database. 100 IQGAP3 expression-related genes were obtained using the GEPIA2 (http://gepia2.cancer-pku.cn/) [25] tool combined with TCGA data, and several genes were selected for validation by coexpression in the TIMER2 database. Functional analysis was performed on two datasets based on R packages of “limma,” “http://org.Hs.eg/.db,” “Cluster Analyzer,” and “Enrichment Map” and displays the 30 paths with the most significant associations.

## 3. Result

### 3.1. IQGAP3 Expression in Tumor and Normal Samples, According to Different Databases

**Figure 1:**
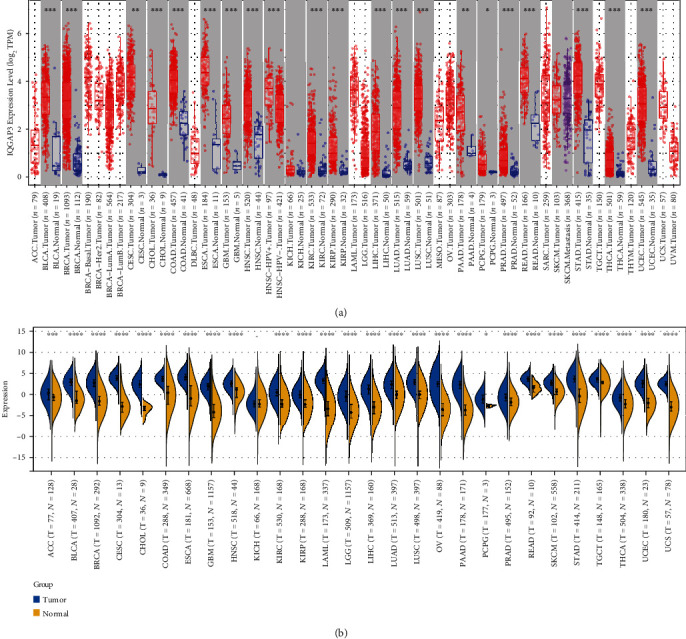
Differential expression of IQGAP3. (a) Comparison of IQGAP3 expression between tumor and normal samples based on TCGA database. (b) Differential expression analysis of IQGAP3 mRNA in different cancers by SangerBox 3.0 based on TCGA and GTEx databases. ^∗^*P* < 0.05, ^∗∗^*P* < 0.01, ^∗∗∗^*P* < 0.001.

We analyzed IQGAP3 expression in TCGA and GTEx databases. First, the expression of IQGAP3 was compared between tumor and normal samples in TCGA database using TMER2. As shown in Figure 1(a), IQGAP3 was upregulated in 20 tumors, including BRCA, UCEC, THCA, GBM, CHOL, CESC, KIRC, HNSC, KIRP, LUSC,LIHC, READ, LUAD, COAD, PAAD, BLCA, PCPG, ESCA, STAD, and PRAD. Due to the small number of some normal tissues in TCGA database or the lack of normal samples, it is not statistically convincing. Therefore, we integrated normal samples from GTEx and databases to match tumor samples from TCGA database to reflect IQGAP3 expression in a more convincing manner. As shown in Figure 1(b), the expression level of IQGAP3 in tumors other than KICH was higher than that in normal tissues.

### 3.2. Expression of IQGAP3 at the Protein Level and Its Location in Cells

**Figure 2:**
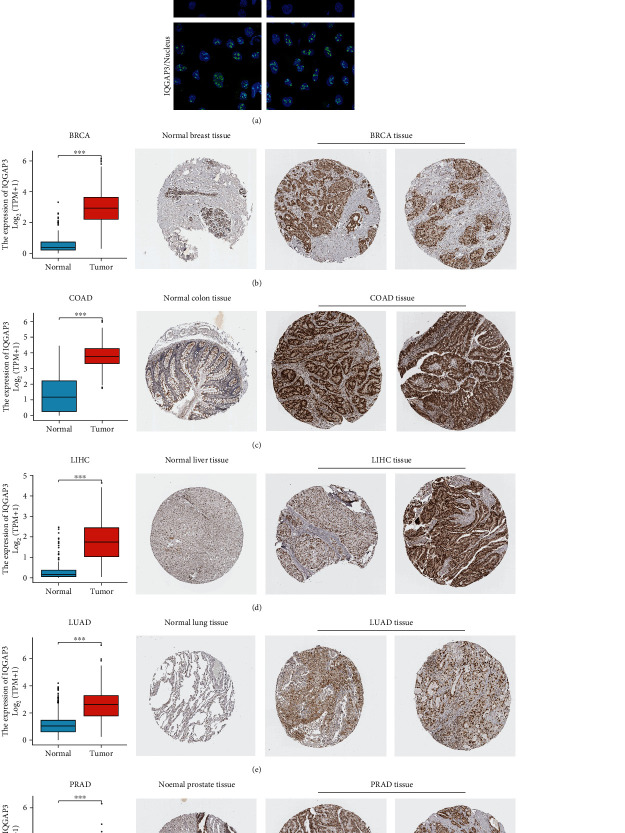
Immunofluorescence of IQGAP3 and comparison of IQGAP3 gene expression in normal and tumor tissues (left) and immunohistochemical images in normal and tumor tissues (right). (a) According to immunofluorescence analysis of skin cancer cell line A-431 and human sarcoma U-2 OS from the HPA database, IQGAP3 protein was almost exclusively located in the nucleoplasm of the cell lines. IQAGP3 mRNA and protein, expressed in (b) BRCA: breast invasive carcinoma, (c) COAD: colon adenocarcinoma, (d) LIHC, (e) LUAD: lung adenocarcinoma, and (f) PRAD: prostate cancer higher than normal tissue. ^∗^*P* < 0.05, ^∗∗^*P* < 0.01, ^∗∗∗^*P* < 0.001.

We used the HPA database to obtain the subcellular location of the IQGAP3 protein. As shown in Figure 2(a), according to the immunofluorescence analysis of human epidermal carcinoma cell line A-431 and human sarcoma U-2 OS cell line, IQGAP3 protein located almost in the nucleoplasm. In addition, based on the HPA database, the IQGAP3 gene expression data of TCGA was compared with the IHC results provided by the HPA database to determine the expression of IQGAP3 at the protein level. The data analysis results of these two databases are consistent. Compared with normal tissues, IQGAP3 protein was significantly overexpressed in tumor tissues of LUAD, BRCA, COAD, LIHC, and PRAD, as shown in Figures 2(b)–2(f).

### 3.3. The Relationship between the Expression Level of QGAP3 and the Survival of Cancer Patients, in Pan-Cancer

**Figure 3:**
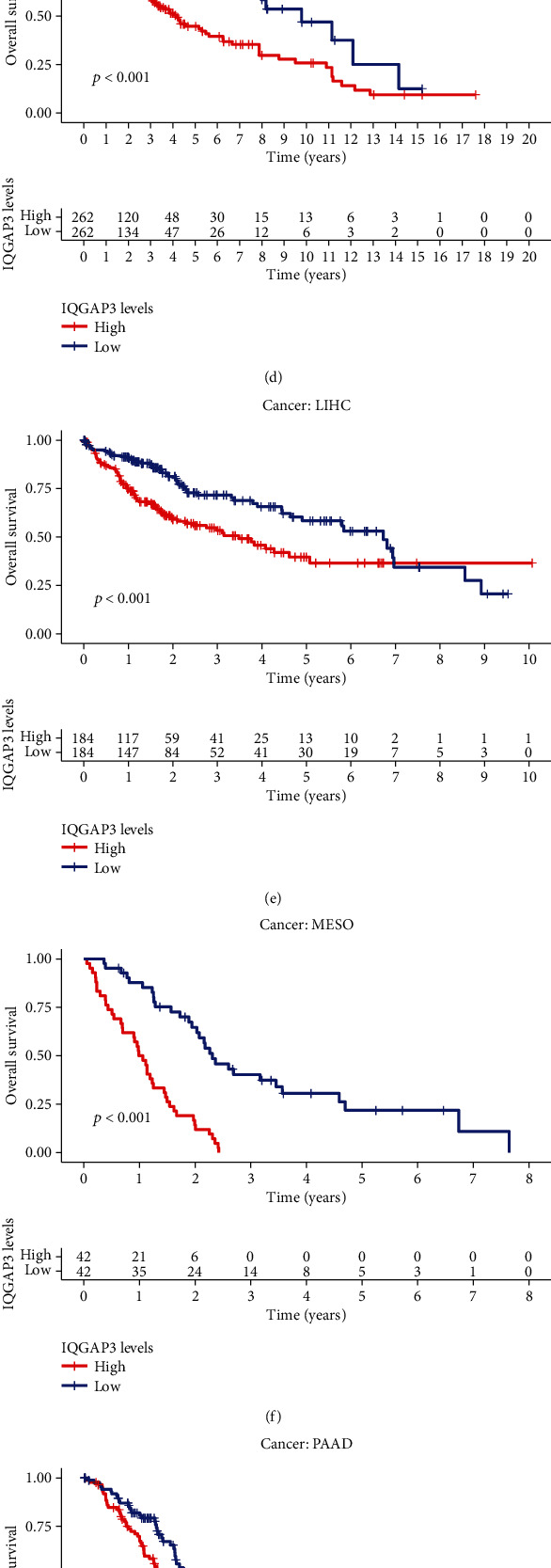
Association between IQGAP3 expression and overall survival (OS). (a–h) The Kaplan-Meier analysis of the association between IQGAP3 expression and OS. (i) Forest plot of OS association in 33 tumors using the univariate Cox hazard analysis.

In pan-cancer, to evaluate the prognostic value of IQGAP3 mRNA expression levels, DSS, OS, PFI, and DFI were analyzed based on TCGA. The results showed that high-expressed IQGAP3 is a risk factor for various cancers, whether DFI, OS, PFI, or DSS. OS Kaplan-Meier curves demonstrated that high-expressed IQGAP3 were associated with poor prognosis in multiple tumors, MESO, ACC, UCEC, LIHC, KIRP, LGG, PAAD, and KIRC Figures 3(a)–3(h). The univariate Cox hazard regression analysis showed that high expression of IQGAP3 mRNA was associated with shorter OS of LIHC, ACC, KIRC, LGG, UCEC, PAAD, KIRP, MESO, PRAD, PCPG, KICH, SKCM, LUAD, and UVM, as shown in Figure 3(i). Furthermore, DSS Kaplan-Meier curves in Supplementary Figures 1A–1I indicated that high-expression levels of IQGAP3 mRNA were associated with poor prognosis in KICH, ACC, PRAD, LIHC, KIRP, MESO, KIRC, UCEC, and LGG. The Cox regression analysis revealed that high expression of IQGAP3 mRNA was a risk factor for ACC, LIHC, KIRP, PCPG, LGG, KIRC, PAAD, KICH, SKCM, UVM, PRAD, UCEC, and MESO, as shown in Supplementary Figure 1J. As shown in Supplementary Figure 2A-F, DFI Kaplan-Meier curves showed that high-expression levels of IQGAP3 mRNA were linked with poor prognosis in KIRP, PAAD, SARC, LUAD, THCA, and PRAD. The Cox regression analysis revealed that high expression of IQGAP3 mRNA was a risk factor for PAAD, LUAD, THCA, KIRP, LIHC, UCEC, SARC, and PRAD, as shown in Supplementary Figure 2P. PFI Kaplan-Meier curves showed that high-expression levels of IQGAP3 mRNA were associated with poor prognosis in UVM, KIRC, ACC, LGG, KICH, THCA, MESO, KIRP, PRAD, LIHC, and SKCM, as shown in Supplementary Figures 3A-K. The Cox regression analysis indicated that high expression of IQGAP3 mRNA was a risk factor for KICH, SKCM, KIRP, LIHC, LGG, PCPG, MESO, PRAD, PAAD, LUAD, UVM, ACC, THCA, KIRC, UCEC, and SARC, as shown in Supplementary Figure 3L.

### 3.4. The Relationship between the Expression Level of QGAP3 and the TNM Stage of Tumor Patients

**Figure 4:**
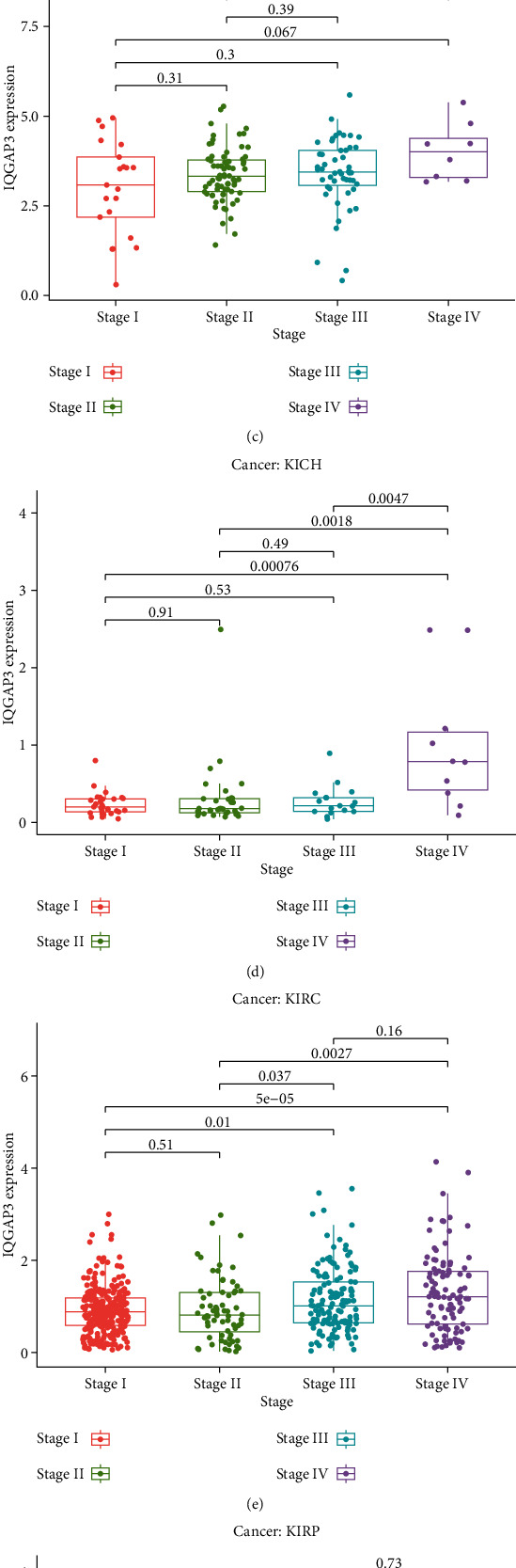
Relationship between IQGAP3 expression and tumor stage (a) adrenocortical carcinoma (ACC), (b) breast invasive carcinoma (BRCA), (c) esophageal carcinoma (ESCA), (d) kidney chromophobe (KICH), (e) kidney renal clear cell carcinoma (KIRC), (f) kidney renal papillary cell carcinoma (KIRP), and (g) thyroid carcinoma (THCA).

We also analyzed the correlation of IQGAP3 expression in tumor stage and found that IQGAP3 expression was significantly correlated with tumor stage in multiple cancers, including BRCA, ACC, ESCA, THCA, KIRP, and KICH, as shown in Figures 4(a)–4(g). Notably, there were significant differences in the expression of IQGAP3, at different stages, especially between the first and fourth stages. Interestingly, the expression level of IQGAP3 increased as the patient's tumor stage increased.

### 3.5. Gene Mutation Analysis Based on the cBioPortal Database, regarding the IQGAP3 Gene

**Figure 5:**
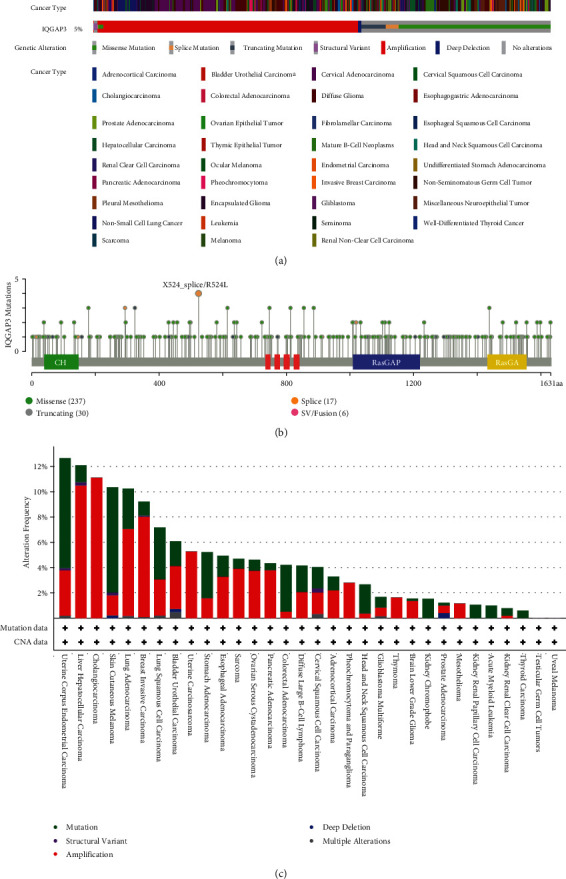
Mutation map of IQGAP3 in cBioPortal-based pan-cancer. (a) IQGAP3 alteration type, (b) mutation site, and (c) alteration frequency in different cancer types.

Using cBioPortal based on TCGA database (10967 samples from 32 studies), mutations in IQGAP3 were analyzed. As displayed in Figure 5(a), in IQGAP3, the total frequency of change is 5%. In addition, the detailed mutation sites are shown in Figure 5(b). In IQGAP3, 290 mutation sites (including 237 missense mutations, 30 truncation mutations, 17 splice mutations, and 6 fusion mutations) were found, located between amino acids 0-163, of which X524_splice/R524L had the highest mutation frequency. In addition, Figure 5(c) shows changes in various cancer types. Among the 32 cancer types, UCEC had the highest frequency of total changes and mutations (>8%), and LIHC and CHOL had the highest frequencies of amplification changes (>10%).

### 3.6. IQGAP3 CNVs in 33 Cancer Types Based on the GSCA Database

**Figure 6:**
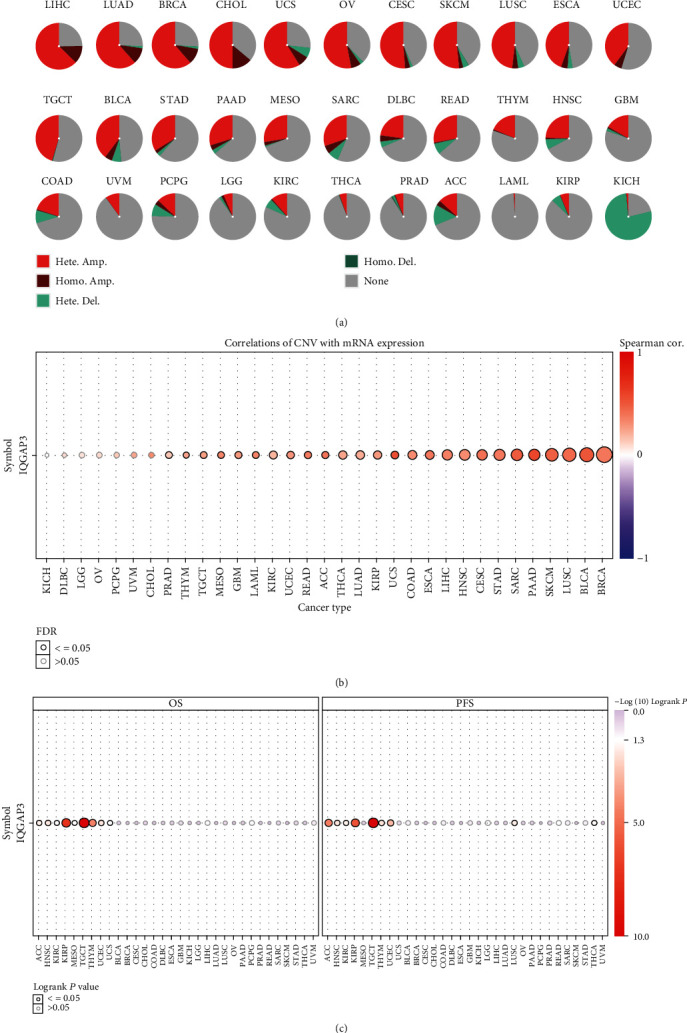
GSCA-based CNV profiling of IQGAP3 in pan-cancer. (a) Deletion/amplification of IQGAP3 heterozygous/homozygous CNVs in various cancer types. (b) Correlation of CNV and IQGAP3 mRNA expression in various cancers. (c) Survival differences between CNV and wild-type groups in pan-cancer type groups.

As shown in Figure 6(a), overall, all tumors except KICH were predominantly amplified and predominantly heterozygous. Among CESC, BRCA, LUAD, UVM, OV, STAD, and LIHC, the heterozygous amplification rate (>50%), the highest heterozygous amplification rate (>62%) in LIHC, and the highest pure sum amplification rate (>13%) were found in CHOL, but KICH had the highest deletion heterozygous rate (>77%). In Supplementary Table 2, the detailed ratios of CNV types in each cancer were shown. In addition, the relationship between IQGAP3 mRNA expression and IQGAP3 CNV was statistically significant in multiple tumors, including STAD, LCA, BRCA, SARC, UCS, PAAD, LUSC, LIHC, KIRC, MESO, ESCA, COAD, GBM, KIRP, ACC, LUAD, READ, HNSC, UCEC, SKCM, THCA, LAML, CESC, TGCT, THYM, and PRAD, for a total of 26 tumors, as shown in Figure 6, as detailed in Supplementary Table 3. Prognostic importance of IQGAP3 CNVs in pan-cancer was analyzed using the GSCA database. The results showed that changes in IQGAP3 CNV were statistically significant with OS in 7 tumors and PFS in 8 tumors. As shown in Supplementary Figures 5A-G, wild-type IQGAP3 and deletion types had higher overall survival than amplified types in KIRC, ACC, UCEC, MESO, KIRP, and THYM tumors, with the exception of UCS. However, there was no statistical difference between wild type and deletion type. As shown in Supplementary Figures 5H-O, in KIRP, ACC, THYM, KIRC, UCEC, and THCA, wild-type IQGAP3 had better progression-free survival than amplified type, whereas LUSC was the opposite. Furthermore, the null-type IQGAP3 outperformed the wild type in ACC and THCA.

### 3.7. The Methylation Profile of IQGAP3 and the Relationship between IQGAP3 Expression and M6A Gene Coexpression

**Figure 7:**
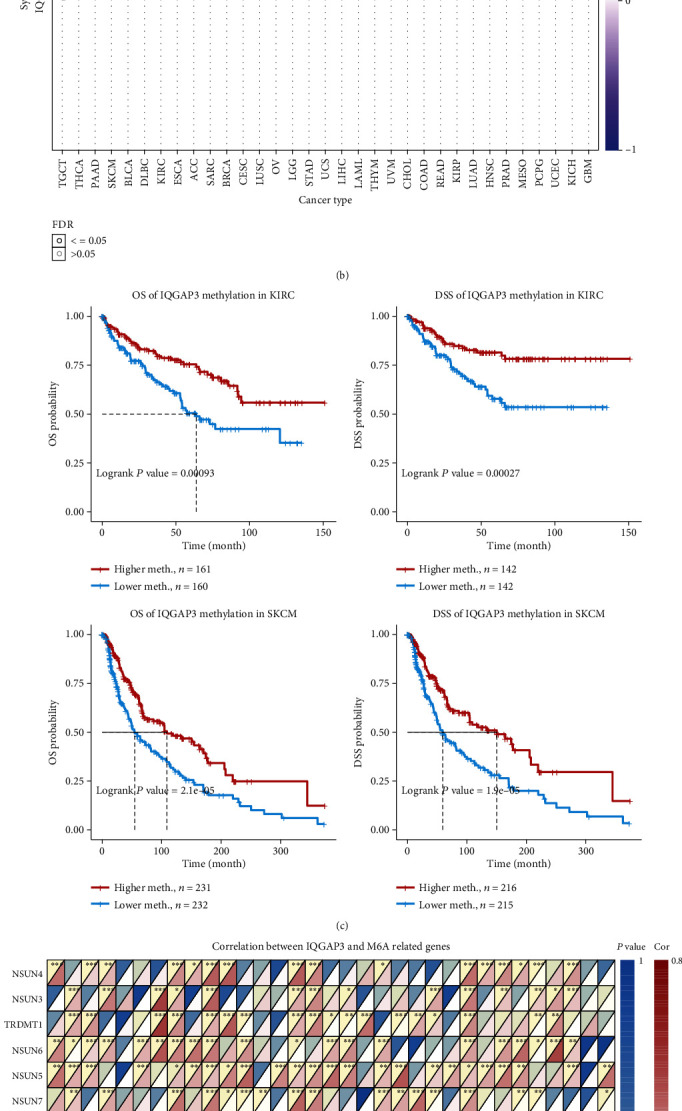
Methylation of IQGAP3 in pan-cancer. (a) Methylation differences of IQGAP3 between tumor and normal tissues. (b) Correlation of methylation with IQGAP3 mRNA expression. (c) Survival difference between IQGAP3 hypermethylation and hypomethylation in KIRC and SKCM. (d) Relationship between M6A-related genes and IQGAP3 mRNA.

Using GSCA methylation sections, we analyzed the differences in IQGAP3 methylation between normal and tumor tissues, and the results are shown in Figure 7(a); in 7 tumors of LUAD, BRCA, LIHC, UCSCC, COAD, KIRC, and PRAD, there were statistical differences, and methylation levels in tumors were lower than in normal tissues. Analysis of IQGAP3 expression and tumor methylation levels revealed that IQGAP3 mRNA expression was negatively correlated with methylation in 28 tumors, as shown in Figure 7(b), and detailed data are in Supplementary Table 4. Analysis of IQGAP3 methylation levels with OS and DSS survival rates showed that both KIRC and SKCM tumors were associated with OS and DSS in their methylation levels, and their hypermethylation levels had higher survival times, and the KM survival curves are shown in Figure 7(c). Additionally, we explored the relationship between IQGAP3 and methylation at the mRNA level and the coexpression of IQGAP3 and M6A-related genes. We found that almost all M6A-related genes were positively correlated with IQGAP3, among which transmethylase-related genes (DNMT3B, DNMT1, and DNMT3A), NOP2, and NSUN2 were highly positively correlated in almost all tumors, as shown in Figure 7(d).

### 3.8. The Relationship between IQGAP3 Expression and Pan-Cancer Immunotherapy and Immune Microenvironment

**Figure 8:**
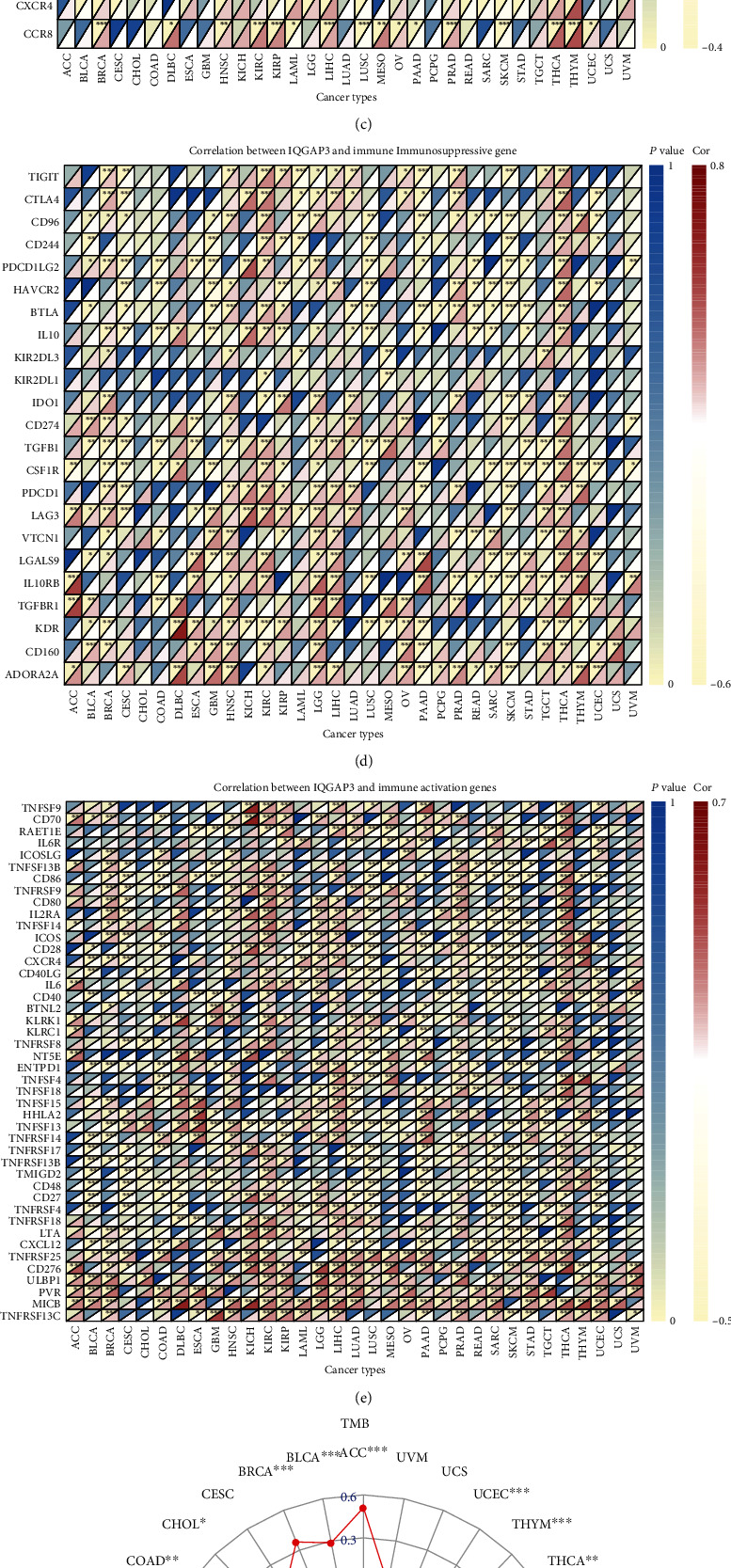
The relationship between IQGAP3 and immune-related genes including (encoding MHC, immune activation, immune suppression, chemokine, and chemokine receptor proteins), TMB, MSI, and MMR based on TCGA database. Heatmap showing that IQGAP3 is associated with (a) MHC-related genes, (b) immune activation-related genes, (c) immunosuppression-related genes, (d) chemokines, (e) chemokine receptor proteins, and (h) MMR correlation between genes. Correlation of IQGAP3 with (f) TMB and (g) MSI.

**Figure 9:**
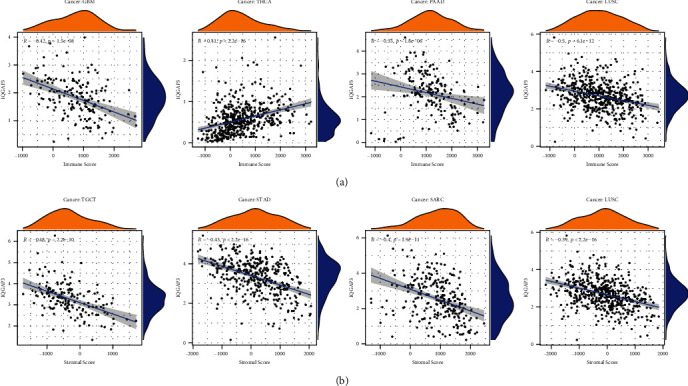
Relationship of IQGAP3 to the immune microenvironment. The correlation of IQGAP3 with (a) immune score and (b) stromal score in different cancers was statistically significant.

First, we explored IQGAP3 expression and coexpression of immune activation, immune suppression, genes encoding MHC, chemokine receptor proteins, and chemokine. As shown in Figures 8(a)–8(e), IQGAP3 expression was statistically significantly correlated with various immune-related genes. Because TMB and MSI are intrinsically associated with immunosuppressive susceptibility, we investigated the correlation of IQGAP3 expression with TMB and MSI in 33 tumors and showed a correlation with TMB in 22 tumors, including BLCA, ACC, KIRC, THCA, SARC, HNSC, LUAD, ESCA, LGG, COAD, PRAD, UCEC, MESO, CHOL, PAAD, BRCA, READ, LUSC, THYM SKCM, KICH, and STAD, except for THYM; other tumors are associated with IQGAP3 which showed a significant positive correlation, as shown in Figure 8(f). In addition, the association between IQGAP3 and MSI was also found in 12 tumors, including LUSC, BLCA, PRAD, CESC, GBM, STAD, LUAD, ACC, ESCA, SARC, UCEC, and DLBC, as shown in Figure 8(g). MSI is usually caused by MMR, so we further explored the coexpression relationship between IQGAP3 mRNA and MMR key genes (MSH2, PMS2, EPCAM, MLH1, and MSH6). We found that IQGAP3 mRNA was significantly positively correlated with MMR signal in almost all tumors, as shown in Figure 8(h), because MSI and TMB are intrinsically linked with immune checkpoint inhibitor susceptibility. The expression of IQGAP3 is related to TMB and MSI of many tumors, further indicating that IQGAP3 may affect tumor growth and development through immunity. Immune microenvironment has important function in tumor development. Therefore, it is critical to investigate further the pan-cancer relationship of IQGAP3 expression with between TME. The ESTIMATE algorithm was used to calculate stromal cell and immune scores for 33 cancers. In addition to THCA and KIRC, the immune scores and stromal scores of other tumors were negatively correlated with IQGAP3 mRNA. The four cancers with high correlation coefficients between immune scores and stromal scores are shown in Figures 9(a) and 9(b). Additional statistically significant tumor stromal and immune scores are shown in Supplementary Figure 6 and Supplementary Figure 7.

### 3.9. Exploring IQGAP3 mRNA Expression Level and Immune Cell Infiltration Based on the TIMER2 Database

**Figure 10:**
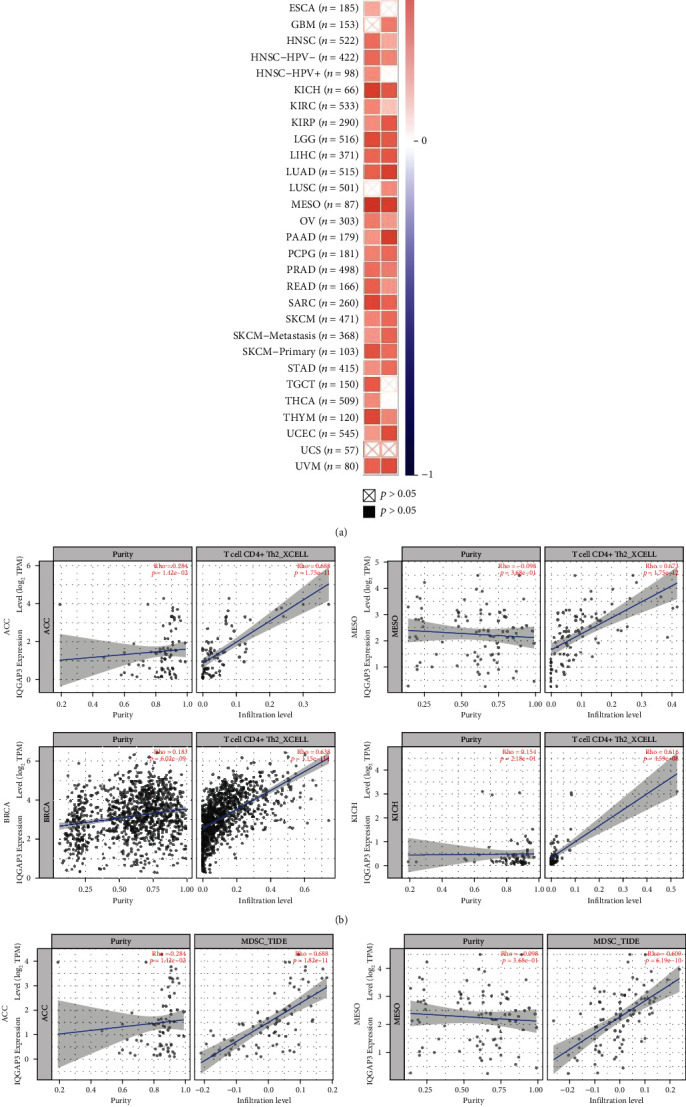
Relationship between immune cells (CD4+ Th2 and MDSCs) and IQGAP3 mRNA expression in pan-cancer based on the TIMER2 database. (a) Heatmap of IQGAP3 mRNA versus CD4+ Th2 and MDSCs infiltration, in pan-cancer. (b) Several tumors with high correlation.

To further understand the role of IQGPA3 in tumor immunity, we explored the relationship between IQGAP3 mRNA expression levels with immune cell infiltration through the TIMER2 database. As shown in Figure 10(a), the expression level of IQGAP3 was positively correlated with CD4 Th2 cells and myeloid-derived suppressor cells (MDSCs) in almost all tumors.  Table 1 shows specific correlation data for IQGAP3 and these two cells. The four tumors with higher correlation coefficients were CD4 Th2 cells and MDSC cells, as shown in Figures 10(b) and 10(c).

### 3.10. Enrichment Analysis of IQGAP3-Related Genes

**Figure 11:**
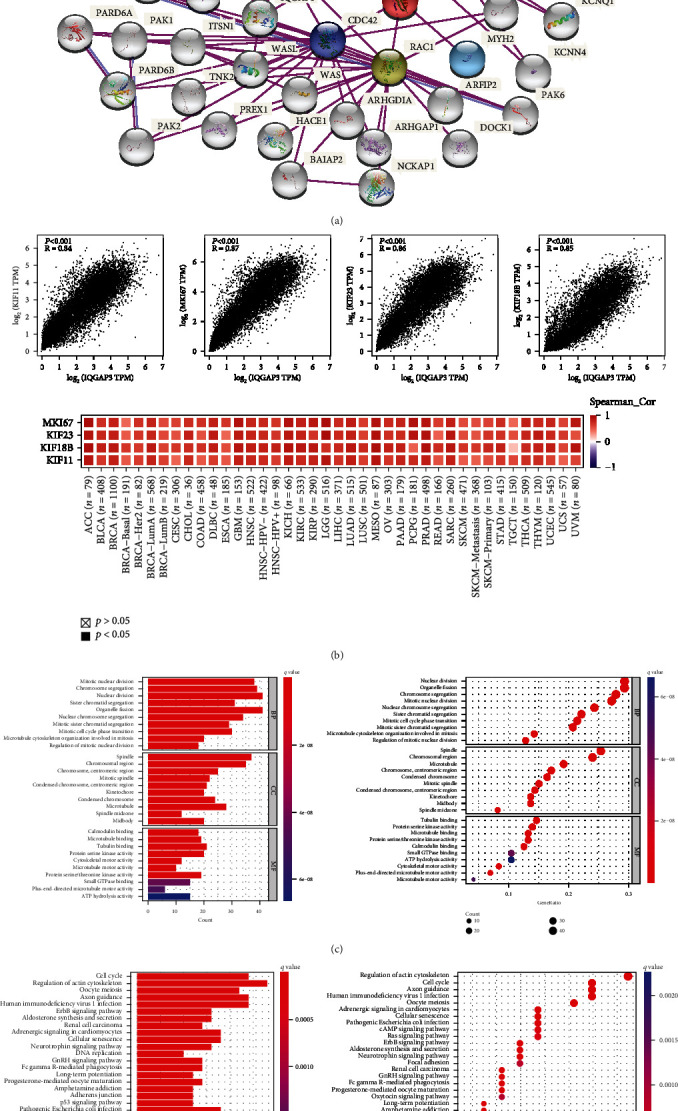
IQGAP3-related gene enrichment analysis. (a) Available experimentally determined IQGAP3-binding proteins were obtained based on the String tool. (b) The top 100 IQGAP3-related genes in TCGA project were also obtained based on GEPIA2. The expression correlation between IQGAP3 and selected target genes (including MKI67, KIF23, KIF8B, and KIF11) was verified by TIMER2, and a heatmap was drawn. (c) The GO pathway analysis based on IQGAP3 binding and interacting genes. (d) The KEGG pathway analysis based on IQGAP3 binding and interacting genes.

To further investigate the molecular mechanism of the IQGAP3 gene in tumorigenesis, we screened 49 experimentally validated IQGAP3-binding proteins based on the String database, as shown in Figure 11(a). To obtain 100 genes related to IQGAP3 expression from TCGA, the GEPIA2 tool was used. Coexpression analysis revealed that KIF11, KIF18B, KIF23, and MKI67 were positively associated with the expression of IQGAP3 in all cancer types presented in the heatmap. We combined two datasets screened from the String database and GEPIA2 database for GO and KEGG enrichment analysis. The GO enrichment analysis of Figure 11(c) shows that IQGAP3-related genomes and major biological processes involved in mitosis and cytoskeleton formation, mainly through small GTPase binding, protein serine/threonine kinase activity, calmodulin binding, and microtubule motility activity, exert molecular function. The KEGG enrichment analysis is shown in Figure 11(d) that IQGAP3 is associated with cell cycle-related pathways, cellular senescence-related pathways, and p53 signaling pathways and affects tumor immunity through immune-related pathways Fc*γ*R-mediated phagocytosis and T cell receptor signaling pathways. Taken together, IQGAP3 likely affects tumor initiation and progression by affecting cell cycle, cellular senescence, and immune-related pathways.

## 4. Discussion

This study is the first multiomics analysis to explore the relation between IQGAP3 and pan-cancer. First, we analyzed the expression of IQGAP3 transcript levels in tumors using data from TCGA through the TMER2 database. Since some tumors of TCGA lack normal samples, we combined the normal samples in GTEx to further verify their expression levels. Second, we validated these transcriptomic results using proteomics based on the HPA database. Third, the significance of IQGAP3 in tumor prognosis was investigated through TCGA database and validated it using the GEO database and analyzed the relationship between IQGAP3 with clinically characteristic tumor TNM staging. Fourth, for the significance of IQGAP3 in CNV, mutation, and other omics, methylation was also determined based on multiple databases. Fifth, the link of IQGAP3 with immune cells, immune microenvironment, and immunotherapy at the multiomics level was analyzed through multiple databases. Finally, we explored the potential pathways of IQGAP3 and functions in tumors through the enrichment analysis. Ultimately, we conclude that IQGAP3 is an effective prognostic biomarker for pan-cancer immune-related therapy. Notably, these valid bioinformatic analyses and repeated validation based on multiomics and multiple databases will ensure the reliability of the results.

Our study showed that IQGAP3 was highly expressed in 29 cancers. IHC analysis confirmed this result at the protein level. Previously, IQGAP3 has been found to be highly expressed in breast cancer [10], colorectal cancer [12], gastric cancer [26], ovarian cancer [27], liver cancer [28], and pancreatic cancer [29]. These studies are consistent with our results and confirm the reliability of our results.

For further assess tumor prognostic value of IQGAP3, the Kaplan-Meier survival analysis and Cox regression analysis was conducted using TCGA data. Our results showed that high IQGAP3 expression was associated with tumor OS, DSS, DFI, or PFI. High IQGAP3 expression was associated with a shorter survival time, which could serve as a poor prognostic factor for tumors. Xu et al. showed that elevated IQGAP3 in urine is a poor prognostic factor for bladder cancer [30]. Oue et al. showed that high expression of IQGAP3 in gastric cancer is a poor prognostic factor [31]. In breast cancer, Hua et al. have confirmed that high expression of IQGAP3 is a poor prognostic factor [32]. These studies all further illustrate the accuracy of our findings. In addition, we found that in certain cancers, IQGAP3 expression was associated with tumor stage, especially between stages 1 and 4. The main tumors included were KICH, KIRP, ESCA, ACC, BRCA, THCA, and KIRC. Clearly, these results indicated that to determine the prognosis of various cancers, IQGAP3 could be used as a biomarker.

As a widely studied epigenetic modification, DNA methylation together with histone modifications functions critically in gene expression regulation and chromatin conformation. We further explored the levels of IQGAP3 methylation in normal and tumor tissues and found that IQGAP3 methylation levels were decreased and statistically significant in 7 tumors. IQGAP3 mRNA expression levels were negatively correlated with IQGAP3 methylation in almost all tumors. Methylation and survival analysis showed better OS in SKCM and KIRC with hypermethylation levels. Therefore, IQGAP3 may affect the survival of SKCM and KIRC by inhibiting the IQGAP3 DNA methylation level. Internal RNA modifications are as well critical to tumors in addition to DNA methylation. Next, we studied the methylation of IQGAP3 mRNA and found that IQGAP3 and M6A-related genes are positively correlated in many tumors. These studies indicate that IQGAP3 may affect the occurrence and development of cancer through methylation.

IQGAP3 was previously reported to be associated with antigen-presenting immune cells [33]. We therefore performed coexpression analysis for assessing the relationship of IQGAP3 expression with immune-related genes and immune activation; the analyzed genes encode MHC, chemokine, chemokine receptor proteins, and immune suppression. It has been found that almost all immune-related genes were coexpressed with IQGAP3. Since MMR deficiency, high MSI and high TMB are crucial for the screening of dominant populations in tumor immunotherapy [34]. IQGAP3 has been shown to be a master regulator of tissue homeostasis and repair [35] The study found that 22 tumors were associated with the presence of TMB, and except for THYM, other tumors were positively associated with IQGAP3. In addition, a correlation between IQGAP3 and MSI was also found in 12 tumors. The coexpression relationship between IQGAP3 mRNA and MMR key genes EPCAM, PMS2, MSH6, MSH2, and MLH1 showed that IQGAP3 mRNA was significantly positively correlated with MMR signal in almost all tumors. This result indicates that IQGAP3 can be used as a new indicator to screen immunotherapy advantaged groups. Since tumor purity is closely related to immunotherapy in the tumor microenvironment [36], we explored the immune score and stromal score in the tumor microenvironment. It was found that high expression of IQGAP3 was associated with lower immune and stromal scores. Therefore, we speculate that IQGAP3 may further affect tumor progression by affecting the tumor immune microenvironment. It has been reported that IQGAP3 can be used as a potential antigen for PRAD mRNA vaccine development and is associated with immunity [33]. In addition, activated T cells upregulated IQGAP3 through PI3K*δ* [37]. To further understand the role of IQGAP3 in tumor immunity, we evaluated the correlation between IQGAP3 expression and various immune cells. It was found that IQGAP3 is associated with a variety of tumor immune cells, especially CD4 Th2 cells and MDSCs cells in almost all tumors; however, the relationship with the immune infiltration of CD4 Th1 cells is not obvious. In cancer, the balance of helper T cells tends to shift from Th1 to Th2 dominance, and a shift in the Th1/Th2 balance has been reported in a variety of tumors, including lung cancer [38], breast cancer [39], cervical cancer [40], and colorectal cancer [41]. MDSCs are a heterogeneous group of bone marrow-derived cells that are precursors of dendritic cells (DCs), granulocytes, or macrophages. They have the ability to significantly suppress immune cell responses and are heavily recruited in tumors. MDSCs are a heterogeneous group of bone marrow-derived cells that are precursors of dendritic cells (DCs), granulocytes, or macrophages. They could greatly inhibit immune cell responses and are heavily recruited in tumors [42]. It has been reported that MDSCs promote angiogenesis, tumor invasion, and metastasis and thus affect tumor development [43]. Therefore, IQGAP3 is likely to lead to immunosuppression and immune escape by stimulating Th2 cells and MDSCs.

In addition, enrichment analysis indicated that IQGAP3 would affect the etiology or pathogenesis of cancer through cell cycle-related pathways, cellular senescence-related pathways, and P53 apoptosis-related pathways, as well as immune responses through T cell receptor signaling pathway and Fc gamma R-mediated phagocytosis. The pathway affects immune infiltration of the tumor microenvironment and thus affects tumor development. As previously reported, Wu et al. identified IQGAP3 as a gene that affects cytoskeletal changes in lung cancer [44]. Leone et al. believed that IQGAP3 is required for normal cell cycle progression and genome stability [45]. Chen et al. found that RAS mediates the inhibition of lymphoma migration and prognosis by BET inhibitors through its negative regulation of IQGAP3 [46]. The above studies further supported that IQGAP3 was highly involved in cancer development and had the potential to be a prognostic biomarker for various cancer types. However, the role of IQGAP3 in more tumors still needs further experiments to verify.

## 5. Conclusions

In conclusion, the current pan-cancer analysis of IQGAP3 revealed that IQGAP3 was differentially expressed in tumor and normal tissues as well as the correlation of IQGAP3 expression with pathological stage, gene mutation, clinical prognosis, and DNA methylation. Furthermore, IQGAP3 expression is associated with MSI, TMB, and immune cell infiltration in different cancer types. Its effects on tumor immunity also vary by tumor type. The current discovery elucidated the function of IQGAP3 in cancer development and tumorigenesis and contributed to a more personalized immunotherapy in the future.

## Figures and Tables

**Table 1:** The relationship between IQGAP3 mRNA and immune cell infiltration.

	MDSC		T cell CD4+ Th2	
	rho	adj.*P*	rho	adj.*P*
ACC (*n* = 79)	0.688	^∗∗∗^	0.688	^∗∗∗^
BLCA (*n* = 408)	0.465	^∗∗∗^	0.483	^∗∗∗^
BRCA (*n* = 1100)	0.495	^∗∗∗^	0.638	^∗∗∗^
BRCA-basal (*n* = 191)	0.167	0.079	0.181	0.053
BRCA-Her2 (*n* = 82)	0.248	0.098	0.348	^∗^
BRCA-LumA (*n* = 568)	0.393	^∗∗∗^	0.467	^∗∗∗^
BRCA-LumB (*n* = 219)	0.406	^∗∗∗^	0.409	^∗∗∗^
CESC (*n* = 306)	0.139	0.063	0.043	0.673
CHOL (*n* = 36)	0.572	^∗∗^	0.386	0.065
COAD (*n* = 458)	0.226	^∗∗∗^	0.205	^∗∗^
DLBC (*n* = 48)	0.197	0.389	0.481	^∗∗^
ESCA (*n* = 185)	0.071	0.541	0.220	^∗^
GBM (*n* = 153)	0.355	^∗∗∗^	0.124	0.291
HNSC (*n* = 522)	0.217	^∗∗∗^	0.410	^∗∗∗^
HNSC-HPV- (*n* = 422)	0.324	^∗∗∗^	0.417	^∗∗∗^
HNSC-HPV+ (n =98)	0.035	0.883	0.306	^∗^
KICH (*n* = 66)	0.491	^∗∗∗^	0.616	^∗∗∗^
KIRC (*n* = 533)	0.143	^∗∗^	0.320	^∗∗∗^
KIRP (*n* = 290)	0.492	^∗∗∗^	0.306	^∗∗∗^
LGG (*n* = 516)	0.485	^∗∗∗^	0.553	^∗∗∗^
LIHC (*n* = 371)	0.498	^∗∗∗^	0.435	^∗∗∗^
LUAD (*n* = 515)	0.596	^∗∗∗^	0.461	^∗∗∗^
LUSC (*n* = 501)	0.307	^∗∗∗^	0.066	0.295
MESO (*n* = 87)	0.609	^∗∗∗^	0.673	^∗∗∗^
OV (*n* = 303)	0.260	^∗∗∗^	0.356	^∗∗∗^
PAAD (*n* = 179)	0.606	^∗∗∗^	0.279	^∗∗^
PCPG (*n* = 181)	0.424	^∗∗∗^	0.333	^∗∗∗^
PRAD (*n* = 498)	0.349	^∗∗∗^	0.400	^∗∗∗^
READ (*n* = 166)	0.274	^∗∗^	0.459	^∗∗∗^
SARC (*n* = 260)	0.445	^∗∗∗^	0.580	^∗∗∗^
SKCM (*n* = 471)	0.420	^∗∗∗^	0.320	^∗∗∗^
SKCM-metastasis (*n* = 368)	0.438	^∗∗∗^	0.274	^∗∗∗^
SKCM-primary (*n* = 103)	0.409	^∗∗∗^	0.516	^∗∗∗^
STAD (*n* = 415)	0.409	^∗∗∗^	0.292	^∗∗∗^
TGCT (*n* = 150)	0.075	0.569	0.487	^∗∗∗^
THCA (*n* = 509)	0.018	0.841	0.308	^∗∗∗^
THYM (*n* = 120)	0.325	^∗∗^	0.567	^∗∗∗^
UCEC (*n* = 545)	0.541	^∗∗∗^	0.266	^∗^
UCS (*n* = 57)	0.236	0.196	0.235	0.197
UVM (*n* = 80)	0.537	^∗∗∗^	0.454	^∗∗∗^

## Data Availability

The data used to support the findings of this study are available in public databases that were included within this article.

## Supplementary Materials

Supplementary 1 Figures S1: association between IQGAP3 expression and disease-free survival (DSS). (A-I) Kaplan-Meier analysis of the association between IQGAP3 expression and DSS. (J) Forest plot of DSS association in 33 tumors. Figures S2: association between IQGAP3 expression and disease-free interval (DFI). (A-F) The Kaplan-Meier analysis of the association between IQGAP3 expression and DFI. (G) Forest plot of DFI association in 33 tumors. Figures S3: association between IQGAP3 expression and progression-free interval (PFI). (A-K) The Kaplan-Meier analysis of the association between IQGAP3 expression and PFI. (L) Forest plot of PFI association in 33 tumors. Figure S4: based on the GEO database, the Kaplan-Meier curves of IQGAP3 in (A-B) BLCA, (C) COAD, (D) LGG, (E-F) LUAD, and (G) OV were significant. Figure S5: (A-G) correlation of IQGAP3 CNV with overall disease survival (OS). (H-O) correlation of IQGAP3 CNV with progression-free survival (PFS). Figure S6: correlation of IQGAP3 with immune scores in the tumor microenvironment. Figure S7: correlation of IQGAP3 with stromal scores in the tumor microenvironment.

Supplementary 2 Table S1: details of 33 cancer types. Table S2: detailed proportion of IQGAP3 CNV types in each cancer type. Table S3: correlation of IQGAP3 CNVs with mRNA expression in various cancer types. Table S4: correlation between IQGAP3 methylation and mRNA expression in each cancer type.
