# Supplementary material for: Comprehensive Multiomics Analysis Identified IQGAP3 as a Potential Prognostic Marker in Pan-Cancer
Source: Dis Markers. 2022 Sep 16;2022:4822964. doi: 10.1155/2022/4822964 (PMC9508463; doi:10.1155/2022/4822964)
Supplement: Supplementary 1 — Figures S1: association between IQGAP3 expression and disease-free survival (DSS). (A-I) Kaplan-Meier analysis of the association between IQGAP3 expression and DSS. (J) Forest plot of DSS association in 33 tumors. Figures S2: association between IQGAP3 expression and disease-free interval (DFI). (A-F) The Kaplan-Meier analysis of the association between IQGAP3 expression and DFI. (G) Forest plot of DFI association in 33 tumors. Figures S3: association between IQGAP3 expression and progression-free interval (PFI). (A-K) The Kaplan-Meier analysis of the association between IQGAP3 expression and PFI. (L) Forest plot of PFI association in 33 tumors. Figure S4: based on the GEO database, the Kaplan-Meier curves of IQGAP3 in (A-B) BLCA, (C) COAD, (D) LGG, (E-F) LUAD, and (G) OV were significant. Figure S5: (A-G) correlation of IQGAP3 CNV with overall disease survival (OS). (H-O) correlation of IQGAP3 CNV with progression-free survival (PFS). Figure S6: correlation of IQGAP3 with immune scores in the tumor microenvironment. Figure S7: correlation of IQGAP3 with stromal scores in the tumor microenvironment. [file 4822964.f1.zip › tables/Supplementary Table 1.docx]

**Cancer abbreviations in the TCGA database**

ACC Adrenocortical carcinoma

BLCA Bladder Urothelial Carcinoma

BRCA Breast invasive carcinoma

CESC Cervical squamous cell carcinoma and endocervical adenocarcinoma

CHOL Cholangiocarcinoma

COAD Colon adenocarcinoma

DLBC Lymphoid Neoplasm Diffuse Large B-cell Lymphoma

ESCA Esophageal carcinoma

GBM Glioblastoma multiforme

HNSC Head and Neck squamous cell carcinoma

KICH Kidney Chromophobe

KIRC Kidney renal clear cell carcinoma

KIRP Kidney renal papillary cell carcinoma

LAML Acute Myeloid Leukemia

LGG Brain Lower Grade Glioma

LIHC Liver hepatocellular carcinoma

LUAD Lung adenocarcinoma

LUSC Lung squamous cell carcinoma

MESO Mesothelioma

OV Ovarian serous cystadenocarcinoma

PAAD Pancreatic adenocarcinoma

PCPG Pheochromocytoma and Paraganglioma

PRAD Prostate adenocarcinoma

READ Rectum adenocarcinoma

SARC Sarcoma

SKCM Skin Cutaneous Melanoma

STAD Stomach adenocarcinoma

TGCT Testicular Germ Cell Tumors

THCA Thyroid carcinoma

THYM Thymoma

UCEC Uterine Corpus Endometrial Carcinoma

UCS Uterine Carcinosarcoma

UVM Uveal Melanoma
