# Supplementary material for: Comprehensive Multiomics Analysis Identified IQGAP3 as a Potential Prognostic Marker in Pan-Cancer
Source: Dis Markers. 2022 Sep 16;2022:4822964. doi: 10.1155/2022/4822964 (PMC9508463; doi:10.1155/2022/4822964)
Supplement: Supplementary 1 — Figures S1: association between IQGAP3 expression and disease-free survival (DSS). (A-I) Kaplan-Meier analysis of the association between IQGAP3 expression and DSS. (J) Forest plot of DSS association in 33 tumors. Figures S2: association between IQGAP3 expression and disease-free interval (DFI). (A-F) The Kaplan-Meier analysis of the association between IQGAP3 expression and DFI. (G) Forest plot of DFI association in 33 tumors. Figures S3: association between IQGAP3 expression and progression-free interval (PFI). (A-K) The Kaplan-Meier analysis of the association between IQGAP3 expression and PFI. (L) Forest plot of PFI association in 33 tumors. Figure S4: based on the GEO database, the Kaplan-Meier curves of IQGAP3 in (A-B) BLCA, (C) COAD, (D) LGG, (E-F) LUAD, and (G) OV were significant. Figure S5: (A-G) correlation of IQGAP3 CNV with overall disease survival (OS). (H-O) correlation of IQGAP3 CNV with progression-free survival (PFS). Figure S6: correlation of IQGAP3 with immune scores in the tumor microenvironment. Figure S7: correlation of IQGAP3 with stromal scores in the tumor microenvironment. [file 4822964.f1.zip › tables/Supplementary Table 2.docx]

cancertype symbol a_total d_total a_hete d_hete a_homo d_homo entrez


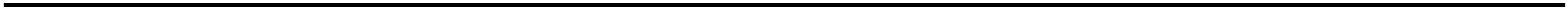

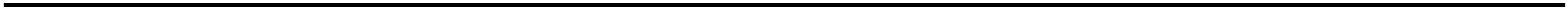

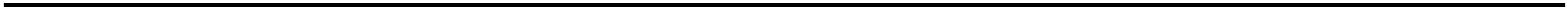


ACC IQGAP3 16.67 14.44 13.33 14.44 3.33 0.00 128239.00
BLCA IQGAP3 44.61 6.86 39.71 6.62 4.90 0.25 128239.00
BRCA IQGAP3 72.22 1.94 61.39 1.94 10.83 0.00 128239.00
CESC IQGAP3 54.58 2.03 51.19 2.03 3.39 0.00 128239.00
CHOL IQGAP3 63.89 0.00 50.00 0.00 13.89 0.00 128239.00
COAD IQGAP3 20.40 9.31 19.96 9.31 0.44 0.00 128239.00
DLBC IQGAP3 27.08 4.17 22.92 4.17 4.17 0.00 128239.00
ESCA IQGAP3 48.91 3.80 44.57 3.80 4.35 0.00 128239.00
 GBM IQGAP3 17.68 2.08 16.81 1.91 0.87 0.17 128239.00
HNSC IQGAP3 25.48 7.28 24.52 7.28 0.96 0.00 128239.00
KICH IQGAP3 1.52 77.27 1.52 77.27 0.00 0.00 128239.00
KIRC IQGAP3 11.93 6.44 11.36 6.44 0.57 0.00 128239.00
KIRP IQGAP3 6.25 6.60 6.25 6.60 0.00 0.00 128239.00
LAML IQGAP3 1.05 0.00 1.05 0.00 0.00 0.00 128239.00
 LGG IQGAP3 7.99 1.95 6.43 1.95 1.56 0.00 128239.00
LIHC IQGAP3 75.14 0.27 62.70 0.27 12.43 0.00 128239.00
LUAD IQGAP3 72.67 1.74 61.63 1.74 11.05 0.00 128239.00
LUSC IQGAP3 52.10 4.59 48.10 4.59 3.99 0.00 128239.00
MESO IQGAP3 29.89 1.15 27.59 1.15 2.30 0.00 128239.00
 OV IQGAP3 59.76 2.59 53.02 2.59 6.74 0.00 128239.00
PAAD IQGAP3 33.15 3.26 29.89 3.26 3.26 0.00 128239.00
PCPG IQGAP3 16.05 8.64 12.96 8.64 3.09 0.00 128239.00
PRAD IQGAP3 6.30 2.64 5.89 1.83 0.41 0.81 128239.00
READ IQGAP3 28.48 7.88 27.88 7.88 0.61 0.00 128239.00
SARC IQGAP3 35.80 7.78 30.35 7.78 5.45 0.00 128239.00
SKCM IQGAP3 54.22 4.36 51.50 4.09 2.72 0.27 128239.00
STAD IQGAP3 35.83 2.72 33.79 2.72 2.04 0.00 128239.00
TGCT IQGAP3 45.33 0.67 45.33 0.67 0.00 0.00 128239.00
THCA IQGAP3 5.41 0.60 5.41 0.60 0.00 0.00 128239.00
THYM IQGAP3 19.51 0.00 18.70 0.00 0.81 0.00 128239.00
UCEC IQGAP3 45.08 0.19 39.89 0.19 5.19 0.00 128239.00
 UCS IQGAP3 66.07 7.14 58.93 7.14 7.14 0.00 128239.00
 UVM IQGAP3 10.00 0.00 10.00 0.00 0.00 0.00 128239.00
