# Supplementary material for: Comprehensive Multiomics Analysis Identified IQGAP3 as a Potential Prognostic Marker in Pan-Cancer
Source: Dis Markers. 2022 Sep 16;2022:4822964. doi: 10.1155/2022/4822964 (PMC9508463; doi:10.1155/2022/4822964)
Supplement: Supplementary 1 — Figures S1: association between IQGAP3 expression and disease-free survival (DSS). (A-I) Kaplan-Meier analysis of the association between IQGAP3 expression and DSS. (J) Forest plot of DSS association in 33 tumors. Figures S2: association between IQGAP3 expression and disease-free interval (DFI). (A-F) The Kaplan-Meier analysis of the association between IQGAP3 expression and DFI. (G) Forest plot of DFI association in 33 tumors. Figures S3: association between IQGAP3 expression and progression-free interval (PFI). (A-K) The Kaplan-Meier analysis of the association between IQGAP3 expression and PFI. (L) Forest plot of PFI association in 33 tumors. Figure S4: based on the GEO database, the Kaplan-Meier curves of IQGAP3 in (A-B) BLCA, (C) COAD, (D) LGG, (E-F) LUAD, and (G) OV were significant. Figure S5: (A-G) correlation of IQGAP3 CNV with overall disease survival (OS). (H-O) correlation of IQGAP3 CNV with progression-free survival (PFS). Figure S6: correlation of IQGAP3 with immune scores in the tumor microenvironment. Figure S7: correlation of IQGAP3 with stromal scores in the tumor microenvironment. [file 4822964.f1.zip › tables/Supplementary Table 3.docx]

cancertype symbol spm fdr entrez


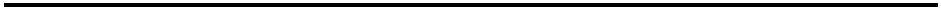

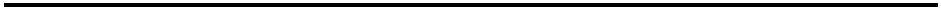

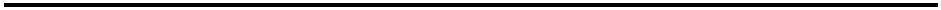


ACC IQGAP3 0.38 0.00 128239.00
BLCA IQGAP3 0.51 0.00 128239.00
BRCA IQGAP3 0.37 0.00 128239.00
CESC IQGAP3 0.40 0.00 128239.00
CHOL IQGAP3 0.32 0.15 128239.00
COAD IQGAP3 0.31 0.00 128239.00
DLBC IQGAP3 0.09 0.79 128239.00
ESCA IQGAP3 0.38 0.00 128239.00
 GBM IQGAP3 0.27 0.00 128239.00
HNSC IQGAP3 0.31 0.00 128239.00
KICH IQGAP3 0.00 0.99 128239.00
KIRC IQGAP3 0.19 0.00 128239.00
KIRP IQGAP3 0.27 0.00 128239.00
LAML IQGAP3 0.33 0.01 128239.00
 LGG IQGAP3 0.07 0.16 128239.00
LIHC IQGAP3 0.35 0.00 128239.00
LUAD IQGAP3 0.22 0.00 128239.00
LUSC IQGAP3 0.43 0.00 128239.00
MESO IQGAP3 0.33 0.01 128239.00
 OV IQGAP3 0.09 0.12 128239.00
PAAD IQGAP3 0.57 0.00 128239.00
PCPG IQGAP3 0.13 0.19 128239.00
PRAD IQGAP3 0.14 0.01 128239.00
READ IQGAP3 0.36 0.00 128239.00
SARC IQGAP3 0.48 0.00 128239.00
SKCM IQGAP3 0.46 0.00 128239.00
STAD IQGAP3 0.37 0.00 128239.00
TGCT IQGAP3 0.25 0.00 128239.00
THCA IQGAP3 0.23 0.00 128239.00
THYM IQGAP3 0.24 0.04 128239.00
UCEC IQGAP3 0.28 0.00 128239.00
 UCS IQGAP3 0.53 0.00 128239.00
 UVM IQGAP3 0.24 0.11 128239.00
