# Supplementary material for: Comprehensive Multiomics Analysis Identified IQGAP3 as a Potential Prognostic Marker in Pan-Cancer
Source: Dis Markers. 2022 Sep 16;2022:4822964. doi: 10.1155/2022/4822964 (PMC9508463; doi:10.1155/2022/4822964)
Supplement: Supplementary 1 — Figures S1: association between IQGAP3 expression and disease-free survival (DSS). (A-I) Kaplan-Meier analysis of the association between IQGAP3 expression and DSS. (J) Forest plot of DSS association in 33 tumors. Figures S2: association between IQGAP3 expression and disease-free interval (DFI). (A-F) The Kaplan-Meier analysis of the association between IQGAP3 expression and DFI. (G) Forest plot of DFI association in 33 tumors. Figures S3: association between IQGAP3 expression and progression-free interval (PFI). (A-K) The Kaplan-Meier analysis of the association between IQGAP3 expression and PFI. (L) Forest plot of PFI association in 33 tumors. Figure S4: based on the GEO database, the Kaplan-Meier curves of IQGAP3 in (A-B) BLCA, (C) COAD, (D) LGG, (E-F) LUAD, and (G) OV were significant. Figure S5: (A-G) correlation of IQGAP3 CNV with overall disease survival (OS). (H-O) correlation of IQGAP3 CNV with progression-free survival (PFS). Figure S6: correlation of IQGAP3 with immune scores in the tumor microenvironment. Figure S7: correlation of IQGAP3 with stromal scores in the tumor microenvironment. [file 4822964.f1.zip › tables/Supplementary Table 4.docx]

cancertype symbol spm fdr entrez


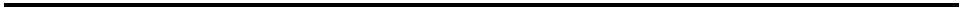

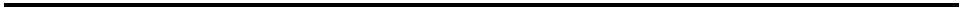

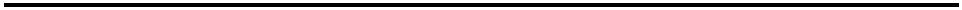


ACC IQGAP3 -0.41 0.00 128239.00
BLCA IQGAP3 -0.31 0.00 128239.00
BRCA IQGAP3 -0.20 0.00 128239.00
CESC IQGAP3 -0.24 0.00 128239.00
CHOL IQGAP3 -0.35 0.04 128239.00
COAD IQGAP3 -0.18 0.00 128239.00
DLBC IQGAP3 -0.55 0.00 128239.00
ESCA IQGAP3 -0.32 0.00 128239.00
 GBM IQGAP3 0.00 0.97 128239.00
HNSC IQGAP3 -0.11 0.01 128239.00
KICH IQGAP3 -0.01 0.91 128239.00
KIRC IQGAP3 -0.29 0.00 128239.00
KIRP IQGAP3 -0.16 0.01 128239.00
LAML IQGAP3 -0.22 0.00 128239.00
 LGG IQGAP3 -0.19 0.00 128239.00
LIHC IQGAP3 -0.19 0.00 128239.00
LUAD IQGAP3 -0.13 0.01 128239.00
LUSC IQGAP3 -0.23 0.00 128239.00
MESO IQGAP3 -0.16 0.14 128239.00
 OV IQGAP3 -0.70 0.04 128239.00
PAAD IQGAP3 -0.45 0.00 128239.00
PCPG IQGAP3 -0.11 0.16 128239.00
PRAD IQGAP3 -0.11 0.02 128239.00
READ IQGAP3 -0.23 0.03 128239.00
SARC IQGAP3 -0.27 0.00 128239.00
SKCM IQGAP3 -0.31 0.00 128239.00
STAD IQGAP3 -0.21 0.00 128239.00
TGCT IQGAP3 -0.57 0.00 128239.00
THCA IQGAP3 -0.37 0.00 128239.00
THYM IQGAP3 -0.24 0.01 128239.00
UCEC IQGAP3 -0.06 0.42 128239.00
 UCS IQGAP3 -0.35 0.01 128239.00
 UVM IQGAP3 -0.27 0.01 128239.00

**迅捷PDF转换器**
