# Supplementary figures and images for: Comprehensive Multiomics Analysis Identified IQGAP3 as a Potential Prognostic Marker in Pan-Cancer
Source: Dis Markers. 2022 Sep 16;2022:4822964. doi: 10.1155/2022/4822964 (PMC9508463; doi:10.1155/2022/4822964)

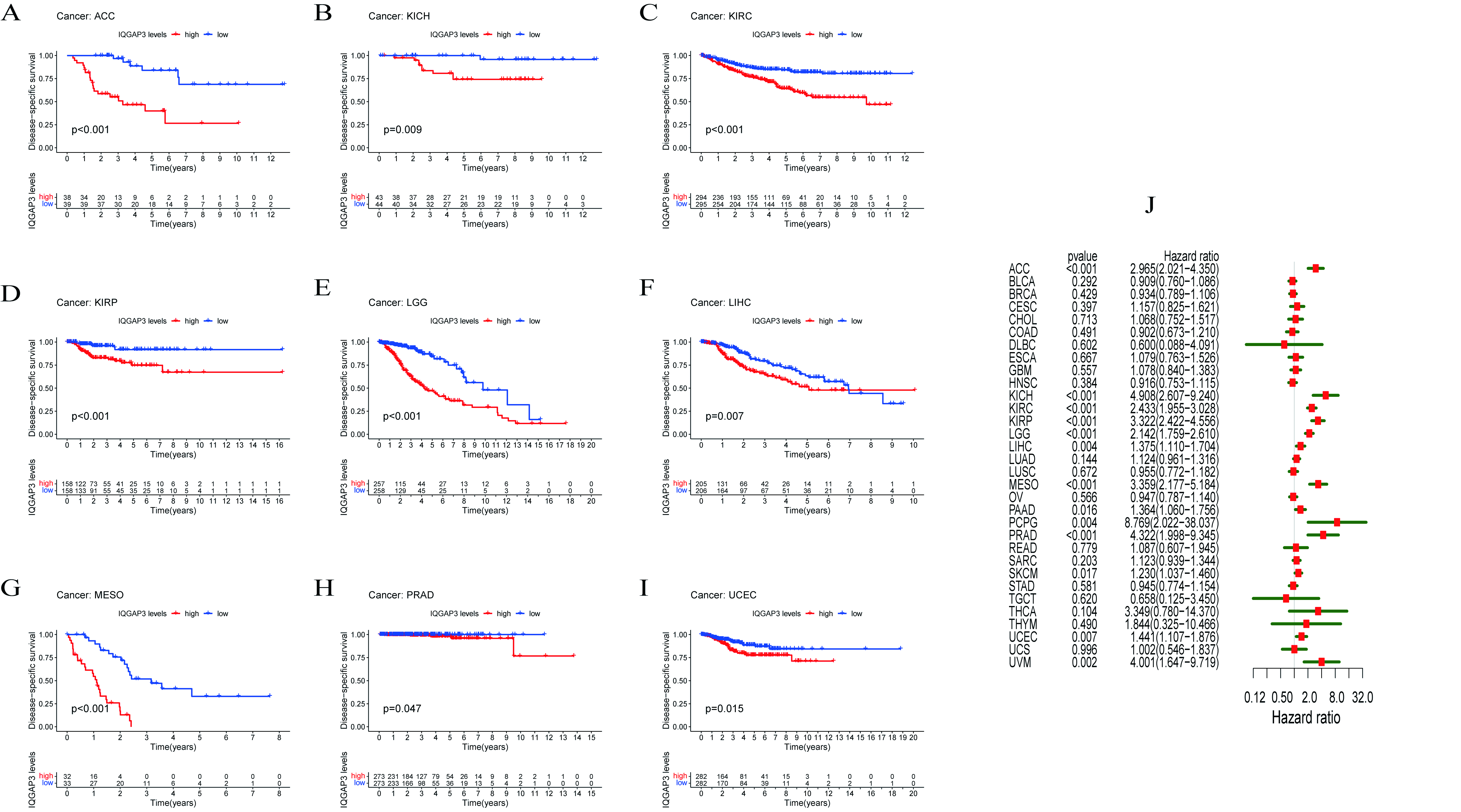

Supplement: Supplementary 2 — Table S1: details of 33 cancer types. Table S2: detailed proportion of IQGAP3 CNV types in each cancer type. Table S3: correlation of IQGAP3 CNVs with mRNA expression in various cancer types. Table S4: correlation between IQGAP3 methylation and mRNA expression in each cancer type. [file 4822964.f2.zip › Supplementary Figures/supplementary Figure 1.jpg]

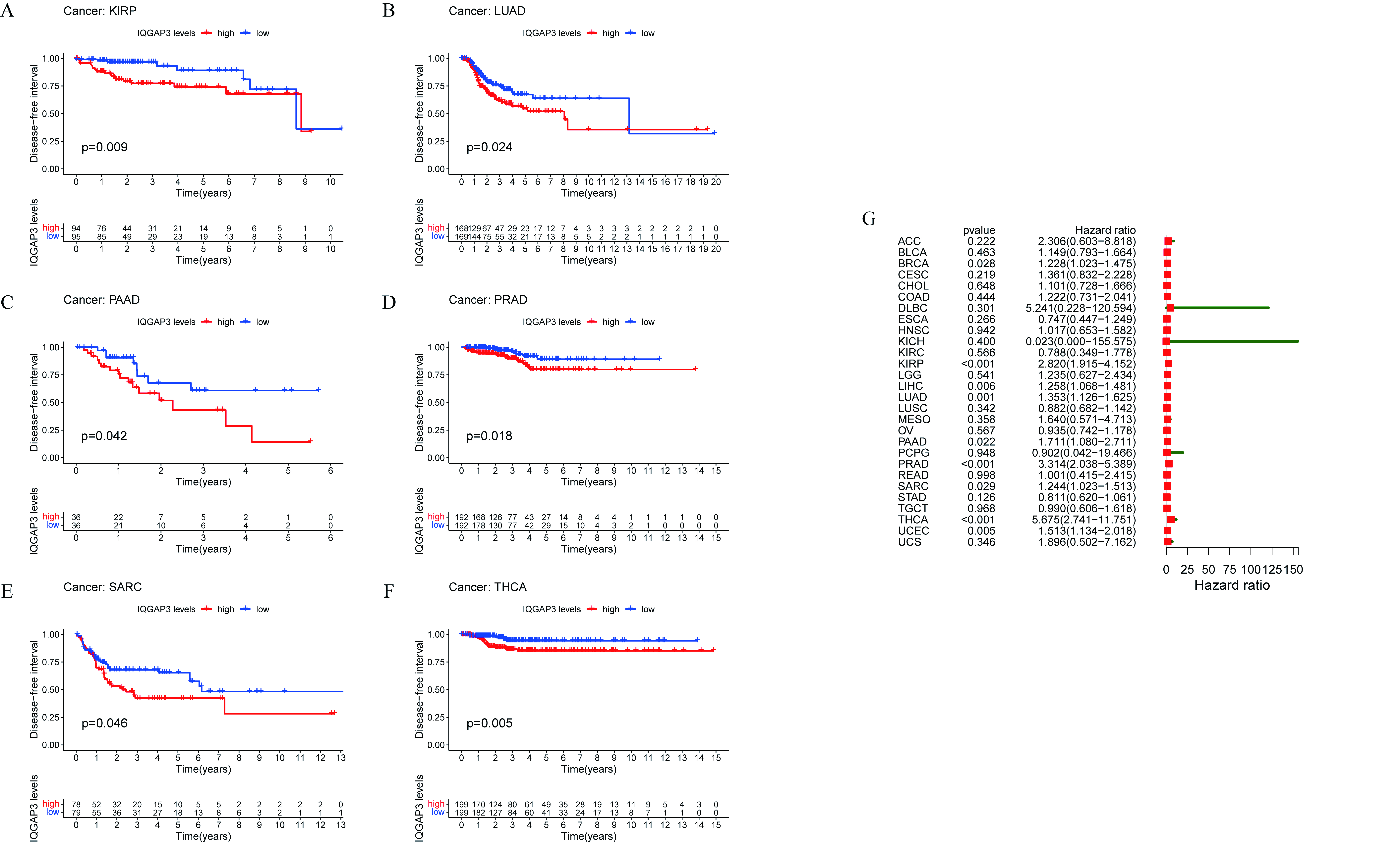

Supplement: Supplementary 2 — Table S1: details of 33 cancer types. Table S2: detailed proportion of IQGAP3 CNV types in each cancer type. Table S3: correlation of IQGAP3 CNVs with mRNA expression in various cancer types. Table S4: correlation between IQGAP3 methylation and mRNA expression in each cancer type. [file 4822964.f2.zip › Supplementary Figures/Supplementary Figure 2.jpg]

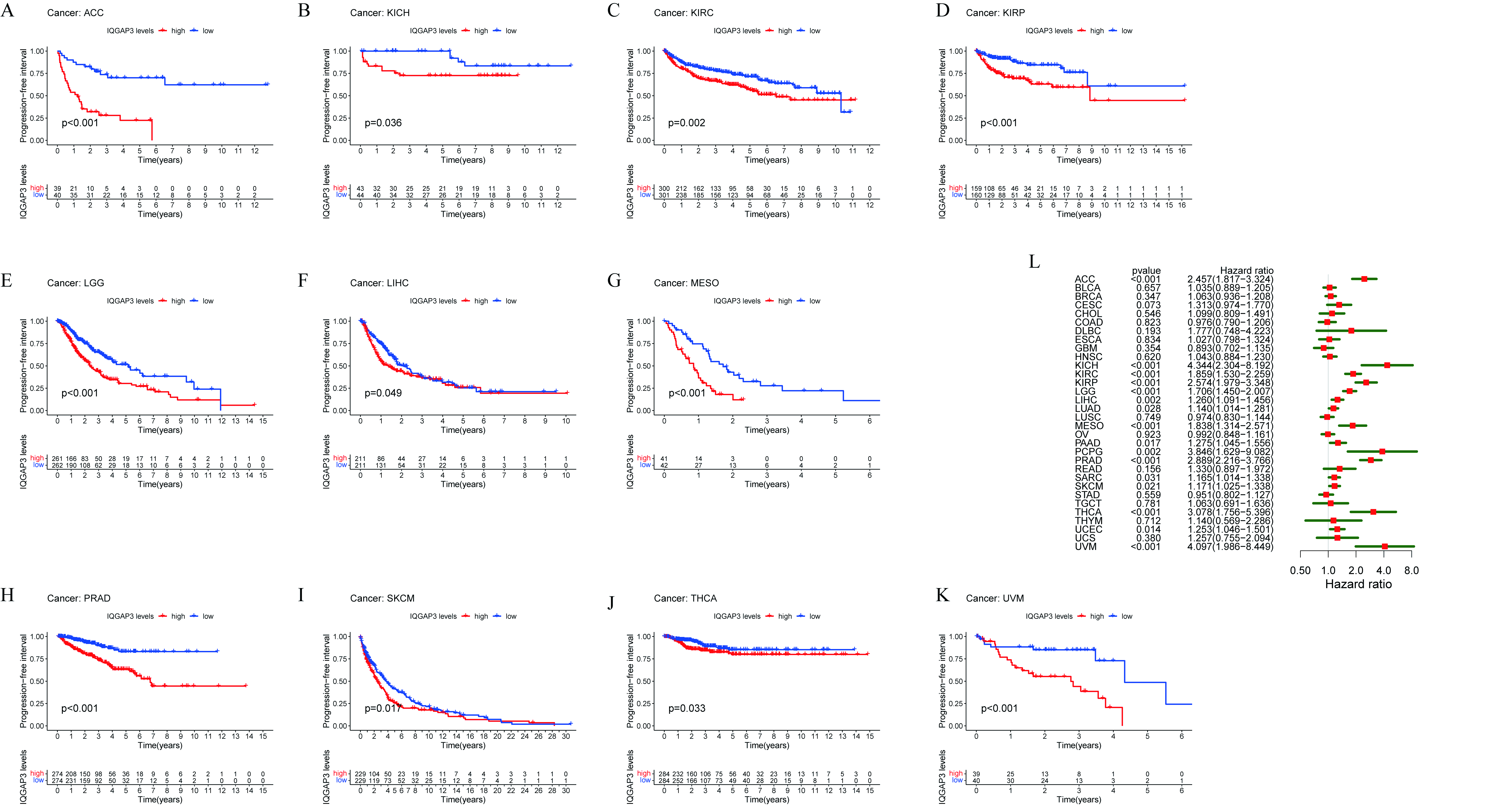

Supplement: Supplementary 2 — Table S1: details of 33 cancer types. Table S2: detailed proportion of IQGAP3 CNV types in each cancer type. Table S3: correlation of IQGAP3 CNVs with mRNA expression in various cancer types. Table S4: correlation between IQGAP3 methylation and mRNA expression in each cancer type. [file 4822964.f2.zip › Supplementary Figures/Supplementary Figure 3.jpg]

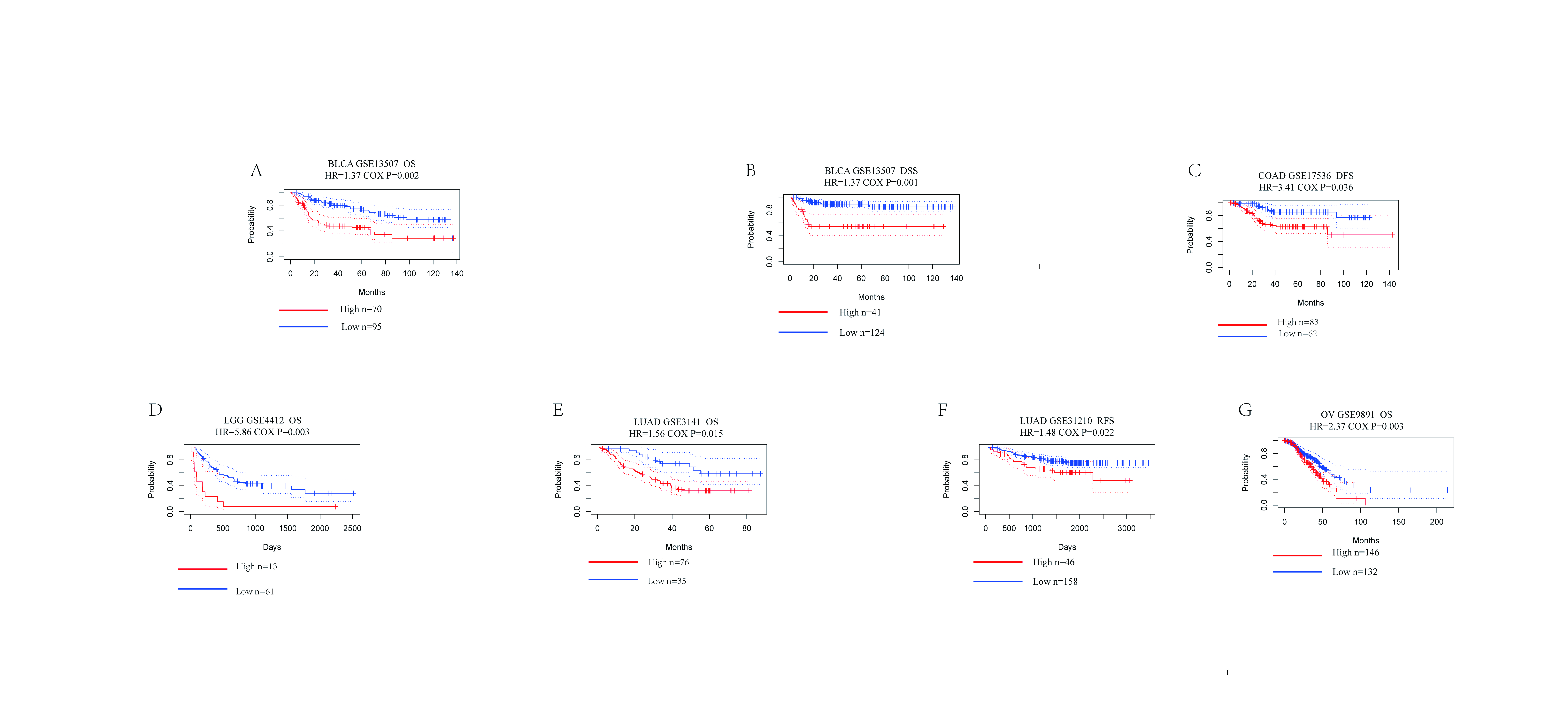

Supplement: Supplementary 2 — Table S1: details of 33 cancer types. Table S2: detailed proportion of IQGAP3 CNV types in each cancer type. Table S3: correlation of IQGAP3 CNVs with mRNA expression in various cancer types. Table S4: correlation between IQGAP3 methylation and mRNA expression in each cancer type. [file 4822964.f2.zip › Supplementary Figures/Supplementary Figure 4.jpg]

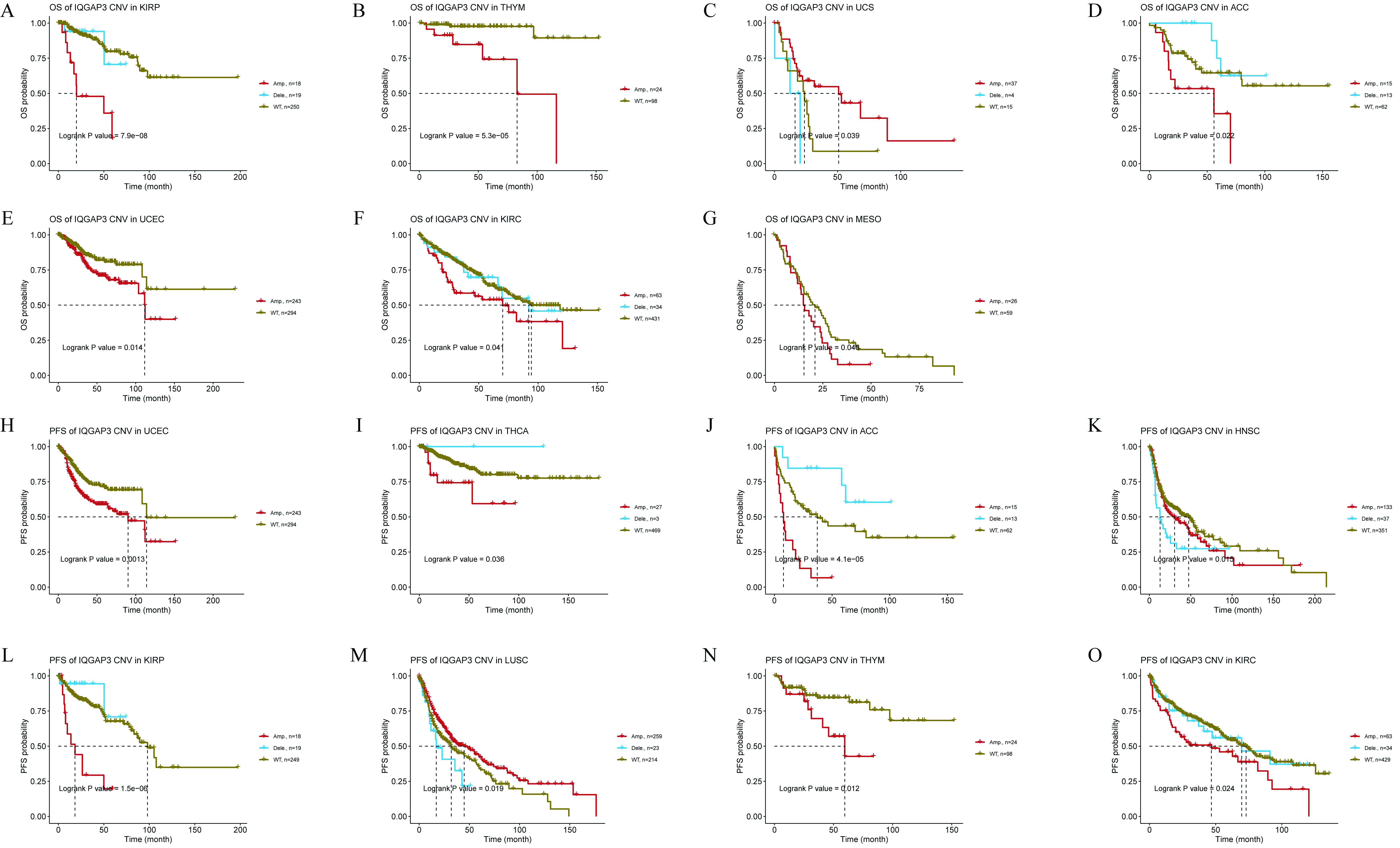

Supplement: Supplementary 2 — Table S1: details of 33 cancer types. Table S2: detailed proportion of IQGAP3 CNV types in each cancer type. Table S3: correlation of IQGAP3 CNVs with mRNA expression in various cancer types. Table S4: correlation between IQGAP3 methylation and mRNA expression in each cancer type. [file 4822964.f2.zip › Supplementary Figures/Supplementary Figure 5.jpg]

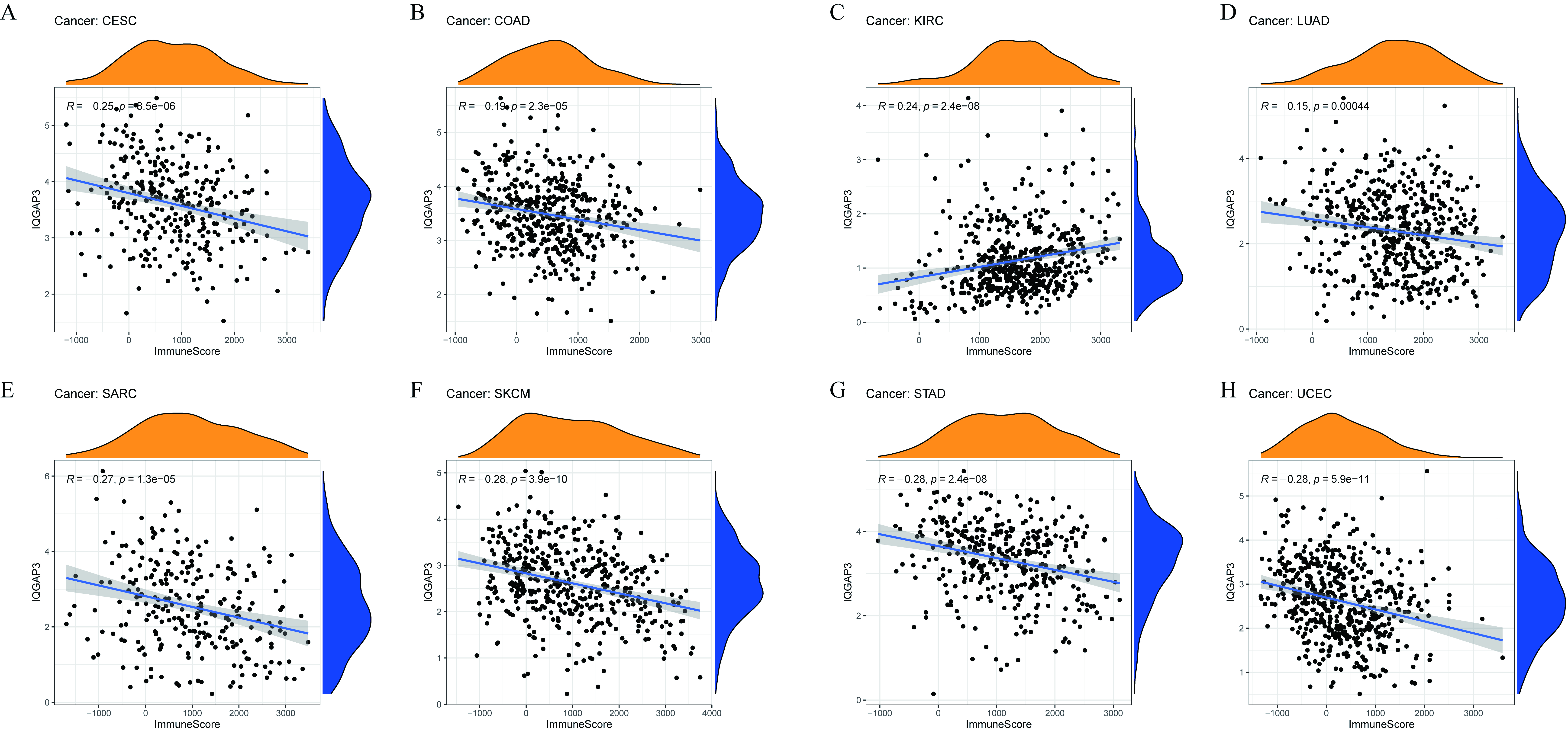

Supplement: Supplementary 2 — Table S1: details of 33 cancer types. Table S2: detailed proportion of IQGAP3 CNV types in each cancer type. Table S3: correlation of IQGAP3 CNVs with mRNA expression in various cancer types. Table S4: correlation between IQGAP3 methylation and mRNA expression in each cancer type. [file 4822964.f2.zip › Supplementary Figures/Supplementary Figure 6.jpg]

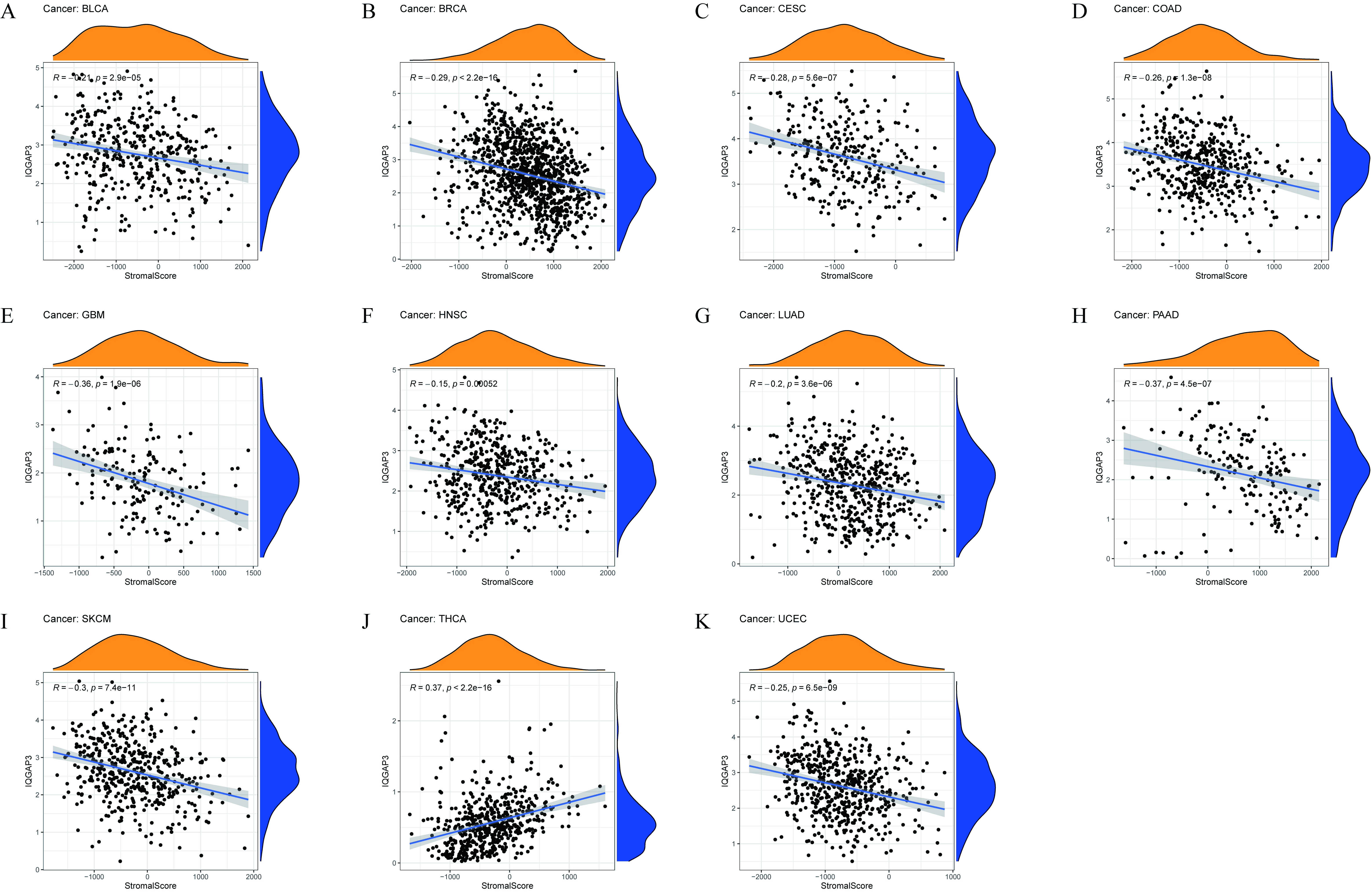

Supplement: Supplementary 2 — Table S1: details of 33 cancer types. Table S2: detailed proportion of IQGAP3 CNV types in each cancer type. Table S3: correlation of IQGAP3 CNVs with mRNA expression in various cancer types. Table S4: correlation between IQGAP3 methylation and mRNA expression in each cancer type. [file 4822964.f2.zip › Supplementary Figures/Supplementary Figure 7.jpg]
